# $\Phi$-Space: continuous phenotyping of single-cell multi-omics data

**DOI:** 10.1186/s13059-025-03755-8

**Published:** 2025-09-30

**Authors:** Jiadong Mao, Yidi Deng, Kim-Anh Lê Cao

**Affiliations:** 1 Melbourne Integrative Genomics, School of Mathematics and Statistics, The University of Melbourne, Parkville, 3010 Victoria Australia; 2 Research School of Finance, Actuarial Studies and Statistics, The Australian National University, Canberra, Australia

**Keywords:** Single-cell, Multi-omics, Reference mapping, Cell type annotation

## Abstract

The prevalence of single-cell multi-omics datasets calls for automated cell type annotation methods that can characterize novel cell states. We developed $\Phi$-Space, a computational framework for the continuous phenotyping of single-cell multi-omics data. We adopt a highly versatile modeling strategy to characterize query cell identity in a low-dimensional phenotype space, defined by reference phenotypes. The phenotype space embedding enables various downstream analyses, including insightful visualizations, clustering, and cell type labeling. $\Phi$-Space is applicable to a wide range analytical tasks beyond cell type transfer. Its ability to model complex phenotypic variations will facilitate biological discoveries from different omics types.

**Supplementary information:**

The online version contains supplementary material available at 10.1186/s13059-025-03755-8.

## Background

Single-cell multi-omics technologies have revolutionized molecular cell biology by providing multi-omic measurement of cells, including genome, transcriptome, epigenome, and proteome. These technologies enable an increasingly refined definition of cell states, which deepens our understanding of cellular heterogeneity and biological complexity [1]. However, the fast generation of population-level and atlas-scale single-cell data has created a huge demand for computationally efficient and statistically reliable annotation of cell populations in emerging datasets [2, 3]. Classifying cells into cell types or states, an analytical task referred to as cell annotation, has been identified as one of the “grand challenges” in single-cell data science [4].

There are two major approaches for cell type annotation: de novo annotation based on marker genes and automated annotation based on reference datasets [4–6].

The de novo approach is well established and consists in applying unsupervised clustering to identify homogeneous cell groups, before manually annotating each group based on their (known) marker genes. However, this approach suffers from two major drawbacks: (i) it is labor-intensive and hence lacks scalability and (ii) it is based on the selection of marker genes and cell type labels that are somewhat arbitrary. These drawbacks result in a lack of scalability and reproducibility [4]. In addition, assigning to a cell cluster a unique cell type label is not appropriate for cells undergoing continuous transition, since discrete clustering often fails to capture within-cluster heterogeneity of developing cells [7].

Automated annotation computationally transfers cell type information from high-quality annotated *reference* datasets to *query* datasets [5]. This approach often uses supervised classification: a classification model (e.g., *k*-nearest neighbor, random forest, or deep neural network) is trained on the reference dataset then assigns a cell type label to each cell in the query single-cell dataset [6, 8–12]. Annotation based on supervised classification has first been developed within omics [13], e.g., scRNA-seq reference and query. Methods for querying scRNA-seq data against bulk references have also been developed to leverage the rich phenotypic information contained in bulk reference atlases [14, 15]. More recently, a cross-omics annotation method was developed [16], where the reference and the query consist of features of different omics types, e.g., scRNA-seq reference and scATAC-seq query.

However, current annotation methods suffer from some common caveats. First, existing methods mainly view cell type annotation as a *hard classification* problem, focusing on accurately predicting the cell types of query cells. These methods lack the power to characterize continuous and transitional cell states. Although methods such as SingleR [12], Seurat V3 [9], and Celltypist [11] do provide some continuous quantification of the predicted cell types, these *soft classification* results remain under-utilized. Second, except scHPL [17] and treeArches [18], which provide hierarchical cell typing, existing methods assign to each cell only one label. Hence, they are unable to jointly model multiple layers of phenotypes (e.g., cell type and sample source). Third, few existing methods (except [12]) can directly use bulk data as reference, due to the much higher proportion of zero counts in scRNA-seq data compared to bulk [15]. As a result, most methods cannot utilize the rich phenotypic information in population-level bulk atlases such as the bulk atlas generated by [14]. Fourth, a cell atlas usually consists of data from multiple experimental batches and studies, and hence suffer from strong and complex batch effects. This poses additional computational challenges for existing annotation methods, since they require the appropriate correction of batch effects within the reference [19]. Lastly, existing methods lack the flexibility to be extended to multi-omics data, resulting in a under-utilization of the increasingly common single-cell multi-omics references [19].

To overcome these limitations, we have developed $\Phi$-Space that uses soft classification to recover the continuous nature of cell states and then use the annotation results as input to downstream analyses. The main innovation of $\Phi$-Space is the phenotype space analysis modeling strategy: the key idea, inspired by a recent work in mathematical statistics [20], is to view soft classification as a dimension reduction to phenotype space and then conduct downstream analyses therein. More specifically, in $\Phi$-Space we assign to each query cell a membership score on a continuous scale for each reference phenotype. Thus, each query cell is continuously characterized in a multi-dimensional *phenotype space*. The phenotype space enables various downstream analyses, including insightful visualizations, clustering but also hard classification.

Compared to analyses in the original omics space, which is the space defined by the original omics features such as gene or protein expressions, we demonstrate that phenotype space analysis is robust against batch effects in both the reference and the query data. The phenotype space analysis makes $\Phi$-Space much more flexible than conventional annotation methods. In particular, $\Phi$-Space can jointly model multiple layers of phenotypes in both bulk and single-cell references. In addition to within- and cross-omics annotation, $\Phi$-Space can also handles multi-omics annotation, where both reference and query contain multimodal measurements (e.g., CITE-seq, gene expression, and surface protein). We show that $\Phi$-Space is highly versatile, and its versatility does not come with a high computational price as $\Phi$-Space is based on linear factor modeling using partial least squares regression (PLS) [21]. Due to PLS’s ability to remove unwanted variation, no additional batch correction nor harmonization of reference and query is needed.

## Results

### Overview of $\Phi$-Space

**Fig. 1:**
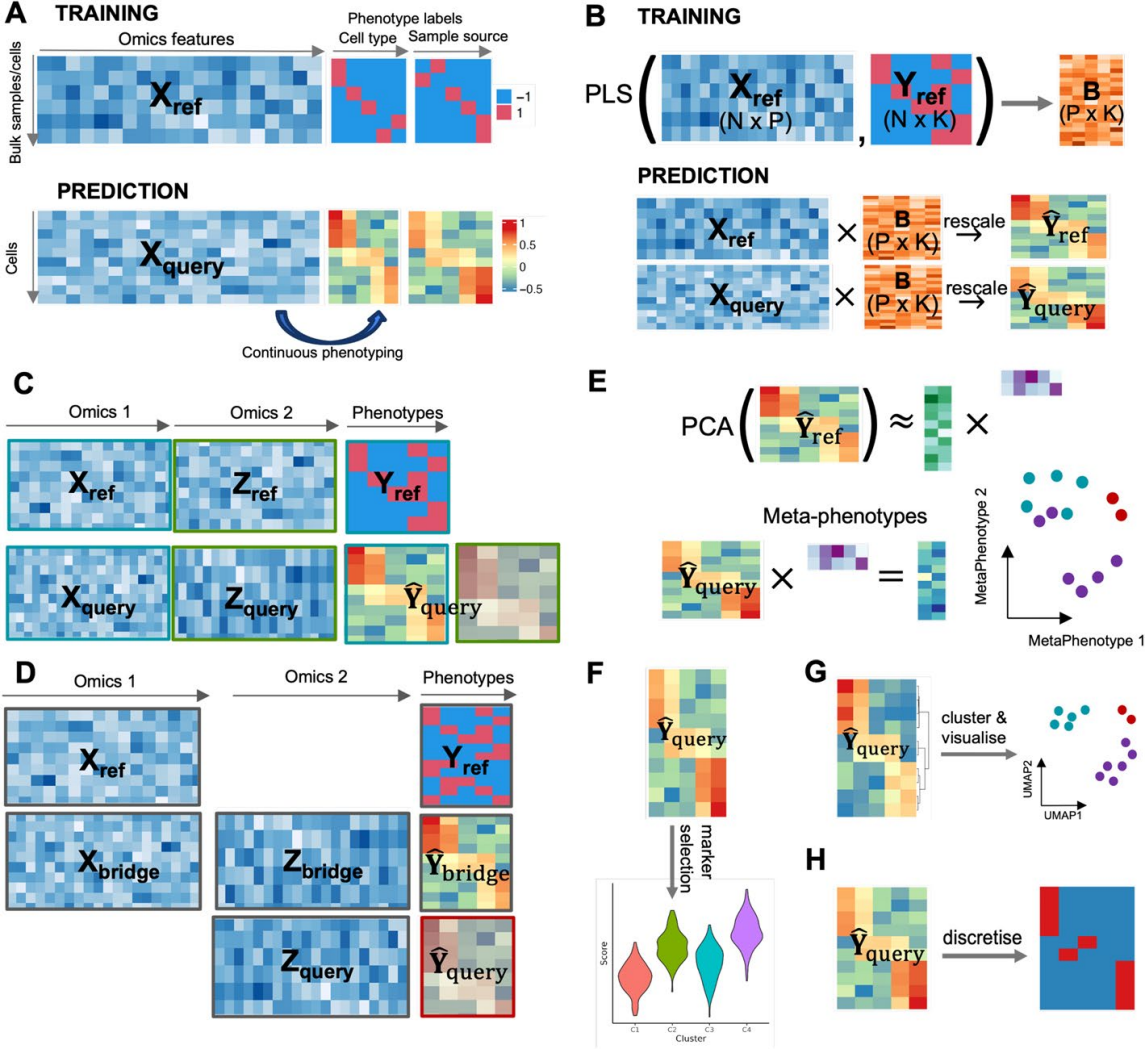
Overview of $\Phi$-Space. **A** $\Phi$-Space continuous phenotyping based on a reference dataset with discrete phenotype labels and a query cell states are then continuously characterized. **B** The core of $\Phi$-Space is partial least squares (PLS) regression. For within-omics annotation, a PLS regression model is trained on reference data $(X_{\text {ref}}, Y_{\text {ref}})$ to compute *B*, a matrix converting omics features to continuous phenotype scores. The phenotype space embeddings of both reference and query are computed as rescaled versions of $X_{\text {ref}} B$ and $X_{\text {query}} B$. **C** $\Phi$-Space multi-omics annotation when both reference and query contain multiple matching modalities. We first conduct within-omics annotation for each modality independently, and then concatenate the phenotype space embeddings derived from different modalities. This multi-omic phenotype space embedding is then used for downstream analysis. **D** $\Phi$-Space cross-omics annotation when reference $X_{\text {ref}}$ and query $Z_{\text {query}}$ contain different omics features. We use a bimodal bridge dataset $(X_{\text {bridge}}, Z_{\text {bridge}})$ sharing omics features with both reference and query: first annotate the bridge cells using the reference modality, i.e., computing $\hat{Y}_{\text {bridge}}$ as rescaled $X_{\text {bridge}} B$; then train a new PLS model to learn the relationship between the other modality $Z_{\text {bridge}}$ and the derived continuous annotation $\hat{Y}_{\text {bridge}}$, resulting in $B_{\text {bridge}}$; finally annotate query data $Z_{\text {query}}$ by computing $\hat{Y}_{\text {query}}$ as rescaled $Z_{\text {query}} B_{\text {bridge}}$. **E**–**H** Downstream analyses based on phenotype space embeddings $\hat{Y}_{\text {ref}}$ and $\hat{Y}_{\text {query}}$. **E** Reference mapping: reduce the dimension of $\hat{Y}_{\text {ref}}$ by principal component analysis (PCA), where each PC is interpreted as a “meta-phenotype,” and then $\hat{Y}_{\text {query}}$ is mapped to this PC space via the loading vectors learned from $\hat{Y}_{\text {ref}}$. **F** Marker selection: given some grouping of query cells (e.g., disease conditions), identify phenotypic markers (e.g., enriched cell types) of each group. **G** Clustering:apply clustering algorithms to $\hat{Y}_{\text {query}}$ to identify biological meaningful cell states in the query. **H** Hard classification: for each query cell, select the highest scored cell type as the predicted cell type for that cell

$\Phi$-Space is a computational framework for continuously phenotyping single-cell datasets based on an annotated bulk or single-cell reference dataset. Figure 1 gives an graphical overview of $\Phi$-Space; see Methods section for an detailed explanation. We present 4 biological case studies to showcase the versatile usability of our $\Phi$-Space framework. These case studies are summarized in Table 1.

**Table 1:** Summary of case studies

Case study	Reference	Phenotypes	Query	Featured analyses	Challenges
DC: developing identity of induced dendritic cells	Stemformatics human DC atlas [23], $N=341$ bulk RNA-seq samples of DC and monocytes from 14 studies	Cell types ($\phi =7$) and cell culture methods ($\phi =4$)	scRNA-seq of $N=33525$ in vitro induced DCs and some control cell types	1) Within-omics annotation	1) Strong batch effects in bulk reference
				2) Phenotype space reference mapping	2) Large differences in sequencing depth and sparsity between bulk and single-cell samples
				3) Phenotype space marker selection	3) Developing cell identities of induced DCs
Perturb-seq: quantifying effects of genetic perturbation on T cell states	DICE (database of immune cell expression, expression quantitative trait loci and epigenomics) [26], $N=1,561$ bulk RNA-seq samples of immune cells	Cell types ($\phi =15$)	Peterb-seq of $N=28453$ T cells with genetic perturbations (70 targets in total)	1) Within-omics annotation	1) Large differences in sequencing depth and sparsity between bulk and single-cell samples
				2) Differential cell state analysis	2) Quantifying effects of genetic perturbations on T cell states
CITE-seq: comparing immune cell composition and severity of COVID-19 patients with different pre-existing autoimmune conditions	CITE-seq PBMC atlas of COVID-19 patients with different severity [27], $N=64733$ cells from 130 donors	Broad ($\phi =18$) and fine ($\phi =51$) cell types and COVID-19 severity ($\phi =9$)	CITE-seq PBMC data from COVID-19 patients with pre-existing autoimmune conditions [28], $N=97499$ from 22 patients	1) Multi-omics annotation	1) Integration of two modalities (RNA and protein) in both reference and query
				2) Phenotype space marker selection	2) Quantitative characterization of COVID-19 severity of autoimmune patients
scATAC-seq: cell type classification within or between omics types	One out of 13 batches of a BMMC scRNA-seq dataset; we use a cross-validation procedure as described in [Sec Sec3] section	Cell types ($\phi =27$)	BMMC scRNA-seq (within-omics annotation) or BMMC scATAC-seq (cross-omics annotation); as described in Methods “[Sec Sec3]” section	1) Cross-omics annotation	1) Differentiating cell types in BMMC
				2) Hard classification	2) Batch effects between reference and query
				3) Phenotype space clustering	3) Accurate cell type transfer from scRNA-seq to scATAC-seq

*N* denotes the number of samples (whether bulk or single cell). Phenotypes refer to reference phenotypes that are transferred to query, where $\phi$ denotes the number of phenotype categories. Featured analyses include types of $\Phi$-Space analyses applied to each case study (see Methods section)

*CITE-seq* cellular indexing of transcriptomes and epitopes by sequencing, *scATAC-seq* single-cell assay for transposase-accessible chromatin with, *PBMC* peripheral blood mononuclear cell, *BMMC* bone marrow mononuclear cell

#### Case study 1: dendritic cells (DC)

We show $\Phi$-Space’s ability to characterize developing cell identity by projecting scRNA-seq data from [22] to a bulk RNA-seq reference atlas generated by [23]. The query scRNA-seq dataset consisted of 33,525 in vitro induced human dendritic cells (DCs) and some in vivo DCs served as control cell types. The bulk reference RNA-seq data consisted of 341 bulk RNA-seq samples of different DC and monocyte subtypes from 14 studies and 10 laboratories. We applied $\Phi$-Space to continuously phenotype the query cells using the 7 cell types and 4 cell culture methods defined in the reference.

The bulk DC atlas [23] is a comprehensive resource for DC biology, including DC subtypes from multiple sample sources (e.g., in vivo, in vitro, ex vivo). Due to the important role of induced DCs in immunotherapeutics [24], this atlas provides a computational resource for validating the molecular characteristics of induced DCs, such as those generated by Rosa et al. [22].

#### Case study 2: Perturb-seq

To fully leverage the refined immune cell states defined in bulk atlases and the intrinsic high signal-to-noise ratio of bulk data, we used $\Phi$-Space to quantify the effects of genetic perturbations on T cell states in a CRISPR activation (CRISPRa) Pertub-seq dataset [25]. CRISPRa Perturb-seq couples gain-of-function CRISPR screening (i.e., the expression of each perturbed gene is enhanced rather than suppressed) with scRNA-seq, allowing us to evaluate the effects of gene activation on gene expression. Over 70 genes were perturbed in this particular study, which contained 28,453 primary human T cells. To evaluate the cell states of these query T cells, we used the DICE bulk atlas containing containing 1561 immune cell samples, where over 1100 were T cell samples consisting of 11 refined cell states [26].

The DICE immune cell atlas [26] contains detailed annotation of T cell states, which serves as a standard reference for T cell biology. By integrating conventional bulk RNA-seq with state-of-the-art Perturb-seq, we effectively designed a $\Phi$-Space-based workflow to quantify the effects of genetic perturbations on altering T cell states.

#### Case study 3: CITE-seq

We illustrate $\Phi$-Space’s ability to integrate different omics modalities in a common phenotype space. We used cellular indexing of transcriptomes and epitopes by sequencing (CITE-seq) data from [27] as the reference and queried CITE-seq data from [28]. CITE-seq provides simultaneous measurement of transcripts (RNA) and surface proteins (ADT, antibody-derived tag) at the single-cell level [29]. The reference atlas contained 64,733 peripheral blood mononuclear cells (PBMCs) from 130 donors with different levels of COVID-19 severity, whereas the query contained 97,499 PBMCs from 22 COVID-19 patients with different types of pre-existing autoimmune diseases. We applied $\Phi$-Space to phenotype the query cells using the 18 broad cell types, 51 fine cell types, and 9 categories of COVID-19 severity.

The reference CITE-seq dataset [27] comes from a large-scale COVID-19 study, which also forms part of the Human Cell Atlas. It contains a large number of patient samples, covering a range of disease severity categories. Mapping the COVID–autoimmunity CITE-seq dataset of Barmada et al. [28] to the reference dataset enables a comprehensive characterization of the immune landscape of COVID-19 patients with pre-existing autoimmune conditions.

#### Case study 4: scATAC-seq

We systematically benchmarked $\Phi$-Space’s ability to transfer cell type labels within or across omics types. We used the bone marrow mononuclear cell (BMMC) 10x multiome dataset from [30]. This bimodal dataset contains matched RNA and assay for transposase-accessible chromatin with sequencing (ATAC) measurements of 69,249 BMMCs from 13 experimental batches, all from healthy donors. The ATAC measurements are available in two resolutions: peaks (computationally inferred open chromatin regions) and gene activity scores (gene level aggregated peaks). Luecken et al. [30] manually annotated the cells, and we use their annotation as ground truth. We designed two cross-validation (CV) procedures:

- For within-omics annotation, we used only the RNA modality of the 10x multiome dataset for CV. In each of the 13 CV iterations, we used 1 of the 13 batches of annotated scRNA-seq data (or its pseudo-bulked version) as the reference and the remaining 12 batches as queries (with ground truth labels hidden).
- For cross-omics annotation, we used both the RNA and ATAC modalities. An additional well annotated BMMC scRNA-seq dataset from a single healthy donor [9] was used as the reference. Then, in each of the 13 CV iterations, we used 1 of the 13 batches of bimodal data as the bridge (Fig. 1D). For the remaining 12 batches, we used only their ATAC modality as scATAC-seq queries (with ground truth labels hidden).

Classification errors, compared to ground truth cell type labels in the query, were then calculated. In the cross-omics annotation case above, since the cell type labels in the reference and those in the multiome data were named according to different conventions, we manually regrouped the labels into common broad cell type labels to make the calculation of classification error possible (see Additional file 1: Tables S1 and S2 for details).

Both the BMMC scRNA-seq dataset [9] and the BMMC multiome dataset [30] are widely used in benchmark studies, due to their well-defined ground truth cell type labels. In addition, the multiome dataset contains multiple batches with unignorable batch effects, which is representative of the real-world experimental designs.

### Continuous characterization of developing cell states

Conventional cell type annotation methods assign a known cell type label in the reference to each query cell. While suitable for identifying well characterized and typical cell types, these methods are under-powered in characterizing transitional or atypical cell identities [8, 9]. In addition, these methods assume each sample or cell has a unique cell type label and thus fail in considering multiple layers of phenotypes. Also, in a typical computational workflow [8, 31], cell type annotation is deployed as a separate step, independent from batch effect removal. This may lead to suboptimal annotation results since phenotypic variation is often confounded with experimental batches (e.g., certain cell types or disease conditions only come from certain batches). Thus, a complete removal of batch effects may entail partial loss of between-phenotype heterogeneity.

To address these problems, $\Phi$-Space provides a holistic approach where batch effects removal is tailored for cell type annotation. Instead of assigning a unique cell type label to each query cell, $\Phi$-Space enables a joint modeling of multiple layers of phenotypes. In addition, the $\Phi$-Space continuous phenotyping results are effective for characterizing transitional cell states. We illustrate these appealing characteristics of $\Phi$-Space using our first case study below. Our analyses described below suggest that $\Phi$-Space can remove strong batch effects in the reference atlas of [23], while preserving enough biology to characterize transitional cell states of the induced dentritic cells (DCs) of [22].

#### $\Phi$-Space selects genes to remove unwanted variation in bulk data

**Fig. 2:**
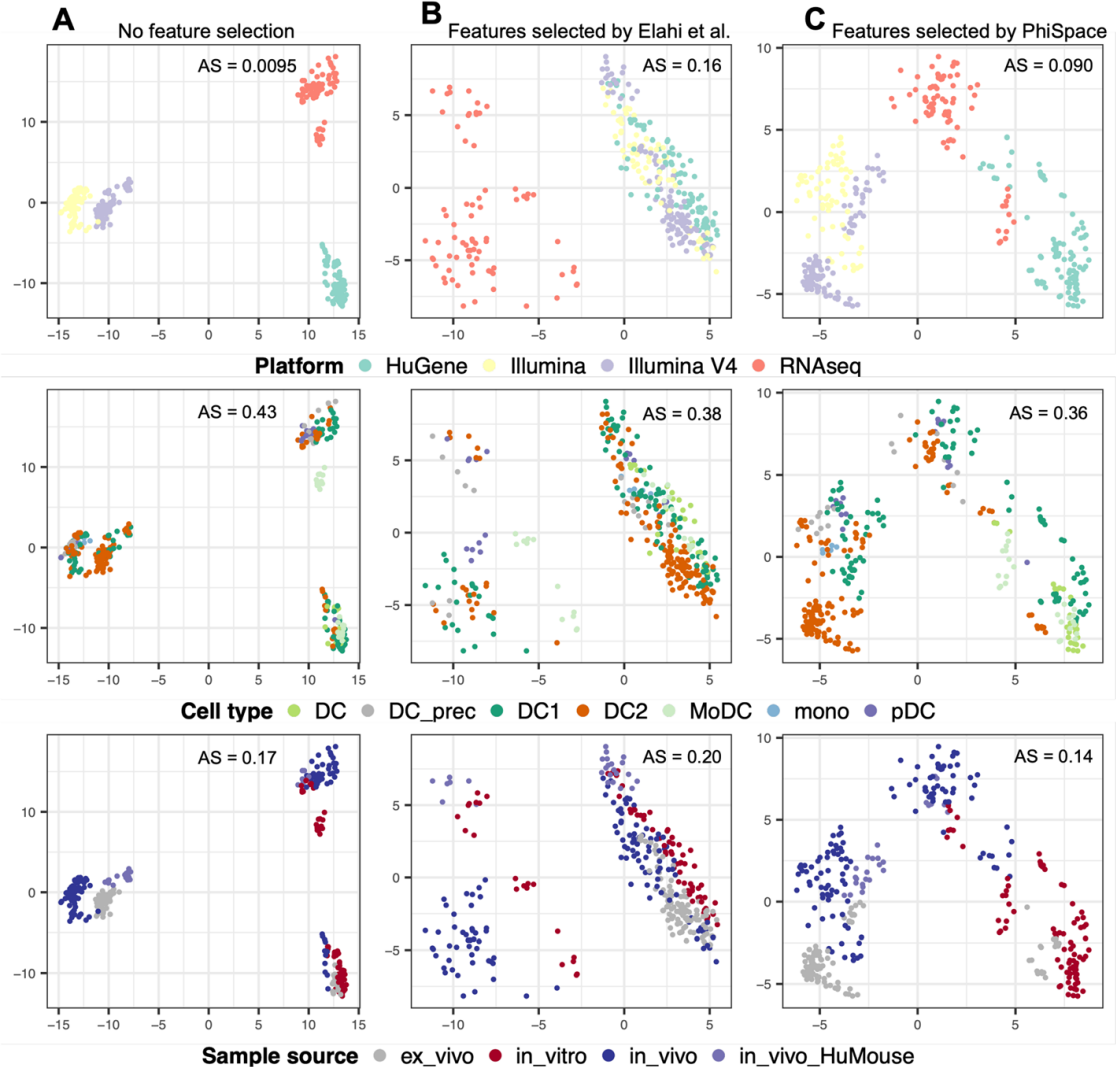
Benchmarking feature selection for building reference atlas. Each column shows all bulk reference samples from [23] viewed from PC1 and PC2 computed using: **A** all features, **B** features selected by [23], and **C** features selected by $\Phi$-Space. The samples are colored by platform, cell type, and sample source. AS: alignment score computed using 20 PCs (see Additional file 2: Section S1.1); larger AS implies better mixing (equivalently poorer separation) of samples from different conditions. Without any gene filtering, the platform effects were the strongest (AS = 0.0095) and the separation of cell types was the poorest (AS = 0.43); the feature selection of [23] significantly removed platform effects (AS = 0.16, best mixing of batches) and had a better separation of cell types (AS = 0.38), but it led to the poorest separation of sample source (AS = 0.20); $\Phi$-Space had the best separation of cell types (AS = 0.36) and sample source (AS = 0.14) by preserving some platform effects that confounds the sample source (AS = 0.090)

Since the DC dataset from [23] contains a large number of 341 samples generated by multiple cell culture methods, it can serve as a reference for benchmarking new in vitro models of DC biology. In particular, it is suitable for testing if the reprogramming method of [22] has successfully cultured induced DCs. However, in the original bulk reference dataset with 16,562 genes, the batch effects caused by the sequencing platform dominates the variation (Fig. 2A). To remove the strong platform effects, [23] applied a feature selection approach introduced by [14] to filter out genes whose variations were mainly explained by the platform, resulting in a total of 2416 genes were kept and the platform effects were much alleviated (Fig. 2B). However, since the platform effects were confounded with the sample source, the differences between, say, in vitro and in vivo samples were also blurred in the PC space. In contrast, $\Phi$-Space selected a smaller subset of 1822 features, which retained some platform effects but, as a result, also preserved a better separation of sample sources (Fig. 2C).

#### $\Phi$-Space phenotype space reference mapping reveals developing cell identity

**Fig. 3:**
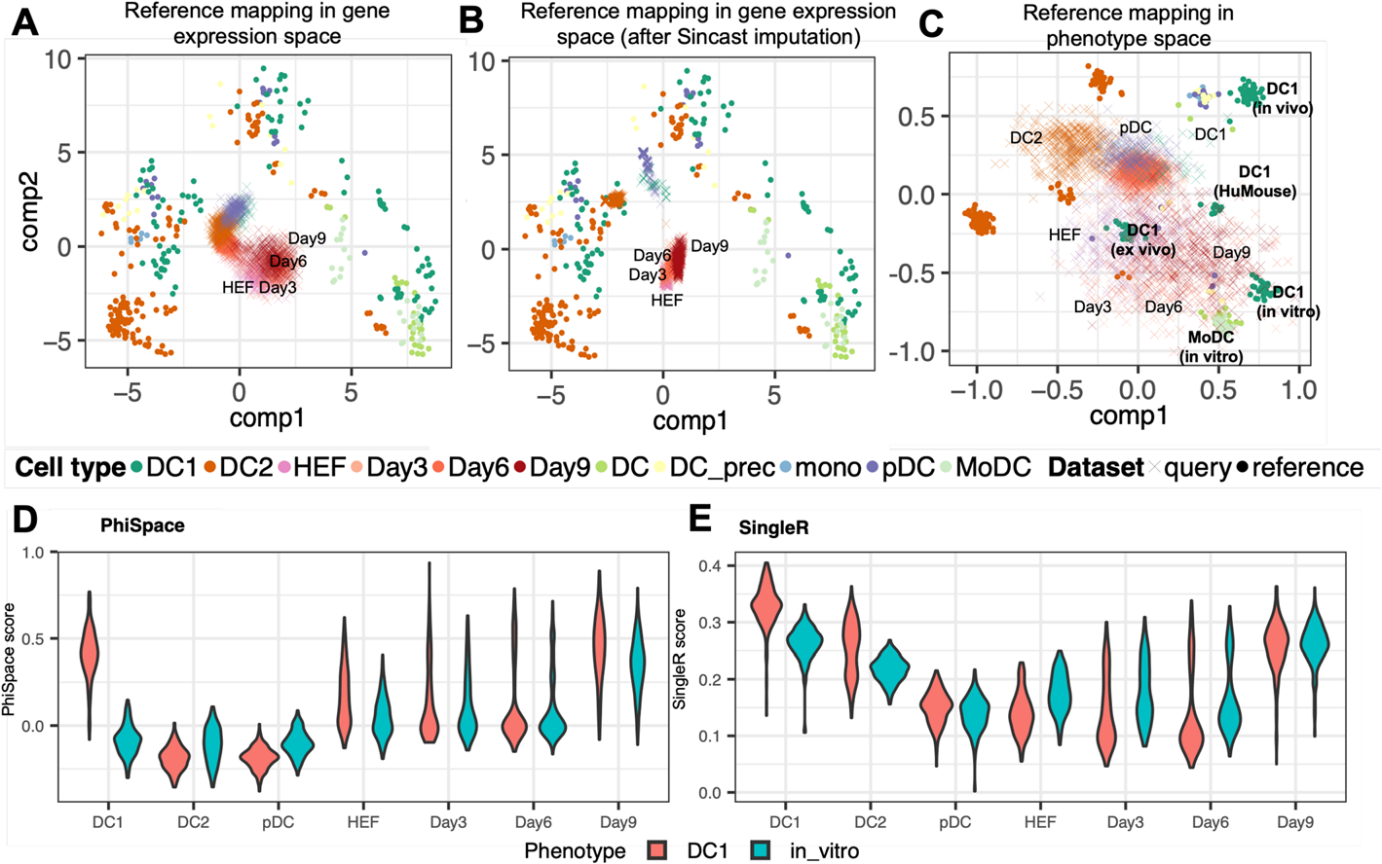
Characterization of developing dendritic cell (DC) identities. **A**–**C** Benchmarking reference mapping in gene space and in phenotype space, showing bulk reference samples ($\bullet$) from [23] and query single cells ($\times$) from [22]. HEFs: human embroyonic fibroblasts; Day3, Day6, and Day9: induced DCs, i.e., HEFs after 3, 6, and 9 days’ reprogramming towards DCs. **A** We first built bulk reference atlas by conducting a PCA of the reference gene expression using genes selected by $\Phi$-Space (depicted in Fig. 2C) and the query gene expression data were mapped to PC1 and PC2 using the bulk reference PCA loadings. The query cells were concentrated around the origin (0, 0) due to the high sparsity of the scRNA-seq gene expression matrix. **B** We then mapped scRNA-seq data imputed using Sincast [15] to the same bulk atlas. The induced DCs were still misaligned with the bulk samples. **C** Lastly we built bulk reference atlas by conducting a PCA of phenotype space embeddings of the bulk reference and the phenotype space embedding of query cells were then projected onto PC1 and PC2. A convergence of reprogrammed HEFs towards in vitro type-1 conventional dendritic cell (DC1) could be observed. **D** and **E** Benchmarking influence of joint modeling on continuous phenotyping: **D** $\Phi$-Space annotation, where a gradual increase of the DC1 and in vitro identities during 9 days of reprogramming could be observed, and where the in vivo non-DC1 control cell types (DC2 and pDC) showed very low DC1 and in vitro scores; **E** SingleR scores computed using genes selected by $\Phi$-Space, where a comparable transition of the induced DCs was observable, but the DC2 cells were assigned overly high DC1 scores

Even though Fig. 2C provides a suitable reference atlas for DC biology, mapping scRNA-seq to this atlas remains challenging. This is due to the technical differences between bulk and single-cell RNA-seq data, including the much higher proportion of zero counts in the single-cell query compared to the bulk reference. A naive PCA-based reference mapping (Additional file 2: Section S1.2) resulted in visualization that is difficult to interpret (Fig. 3A) [15] tackled this problem by proposing an imputation method to reduce the sparsity of the scRNA-seq query. However, Sincast imputation did not significantly improve the interpretability of reference mapping results (Fig. 3B): while the in vivo DC subtypes (DC1, DC2, and pDC) showed improved alignment with their bulk counterparts, the induced DCs (Day3, Day6, and Day9) remained misaligned with bulk samples. This result suggested that imputation might not be sufficient for bridging the gaps between bulk and single-cell RNA-seq data in this case.

Unlike conventional annotation methods, $\Phi$-Space focuses on the phenotype space rather than the gene expression space. This approach enables us to circumvent the harmonization of bulk reference and single-cell query. In $\Phi$-Space, we first compute the PCs of $\hat{Y}_{\text {ref}}$, instead of the gene expression matrix $X_{\text {ref}}$, and then map $\hat{Y}_{\text {query}}$ to these PCs (see Methods “Downstream analyses” section). Figure 3C shows the phenotype space reference mapping results for the DC case. After 3, 6, and 9 days of in vitro reprogramming, the induced DCs converged to the bulk in vitro type-1 conventional dendritic cell (DC1) samples in the reference. In contrast, the DC1s in the query, used by [22] as the control, tended to be closer to their bulk counterparts compared to all other cells. Overall, we observed that the in vivo and induced DCs tended towards two different directions defined by the reference phenotypes. This was confirmed by the heatmap representation of the phenotype space embedding of query cells and a marker gene analysis (Additional file 1: Figs. S1 and S2).

#### $\Phi$-Space can jointly model two layers of phenotypes

In addition to removing unwanted source of variation, $\Phi$-Space’s joint modeling of cell type and sample source yielded more interpretable continuous phenotyping results (Fig. 3D and E). This can be seen by comparing our results to SinlgeR [12], which also provides continuous phenotyping but can only separately model cell type and sample source. From the $\Phi$-Space results (Fig. 3D) we observed a gradual increase of both DC1 and in vitro scores of induced DCs during the reprogramming and a clear distinction of the three control cell types DC1, DC2, and pDC (all in vivo). We then applied SingleR [12] to genes selected by $\Phi$-Space (Fig. 3E), as SingleR is one of the few methods designed to annotate scRNA-seq data using bulk RNA-seq reference. While SingleR provides continuous phenotyping results based on the Spearman correlation between the reference and query samples, it is unable to train a multi-label model to predict the cell type and the sample source simultaneously. Therefore, we trained two SingleR models to separately predict the two layers of phenotypes, cell type and sample source. From the SingleR results (Fig. 3E), we observed a comparable transition from HEF to Day 9 induced DCs. However, the SingleR results suggested that the in vivo DC1 and DC2 in the query had strong in vitro DC1 identity, which was not biologically sensible, suggesting that SingleR was underpowered to jointly model the phenotypic variation pertaining to cell type and sample source.

#### Summary

Through the DC case study, we illustrated how $\Phi$-Space provides a streamlined way for mapping scRNA-seq queries to bulk reference atlases and reveal developing cell identities. We showed that $\Phi$-Space selected biologically meaningful genes to preserve the phenotypic variations in a heterogeneous collection of bulk datasets. To visualize bulk samples and single cells side by side, $\Phi$-Space performs reference mapping using the phenotype space embeddings of reference and query rather than the gene expression. By doing so, our approach does not require explicit harmonization of these two types of data, such as imputation. In addition, $\Phi$-Space successfully modeled two different layers of cell phenotypes, cell type and sample source, yielding better results compared to modeling them independently. In terms of biological insights, our analyses described above confirmed the claim of [22] that their experimental method has reprogrammed HFEs to induced DCs with DC1-like transcriptional profile.

### Quantifying effect sizes of genetic perturbations in Perturb-seq

Perturb-seq has been widely used to quantify the effects of genetic perturbations on cellular gene expression, due to its unique capability to couple CRISPR screening with scRNA-seq [32, 33]. However, since the perturbation of individual target genes often have relatively small effects on the expression of individual target genes, quantifying the effects of genetic perturbations on cell state alterations is challenging. Here we propose a strategy based on $\Phi$-Space to address this challenge. We first annotate perturbed cell states using a reference dataset with $\Phi$-Space. We then examine how individual perturbations affect the phenotype space embedding of perturbed cells compared to unperturbed cells. Since the phenotype space embedding quantifies cell states, we can directly assess the effect sizes of genetic perturbations on cell states.

We quantified the effects of the perturbation of over 70 genes in a CRISPR activation (CRISPRa) screening of human primary T cells [25] with $\Phi$-Space. The DICE (database of immune cell expression, expression quantitative trait loci and epigenomics) bulk RNA-seq atlas [26] was used as the reference to derive T cell states. See [Sec Sec3] section for more details on the dataset.

#### $\Phi$-Space captures nuanced T cell states

**Fig. 4:**
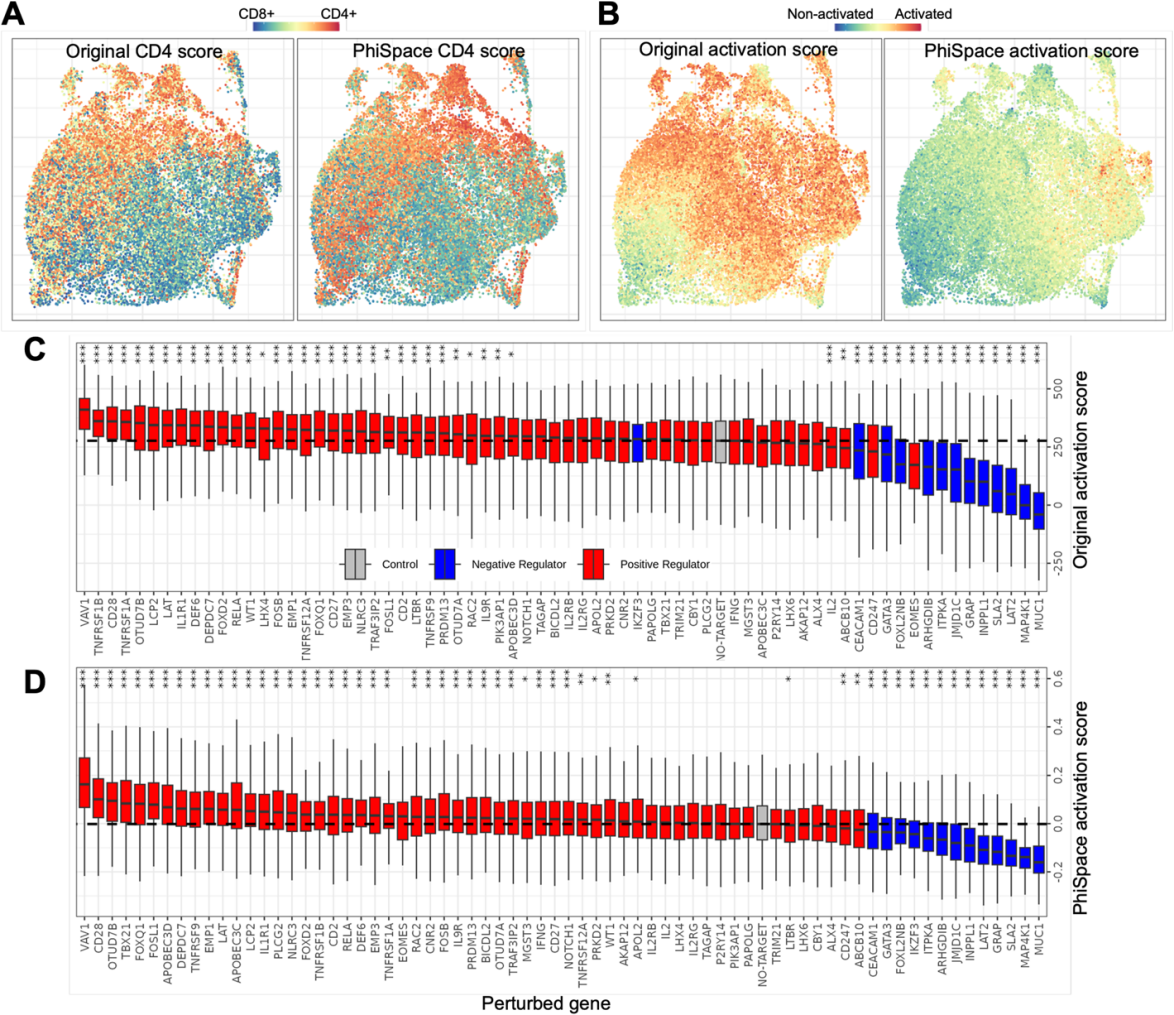
Quantifying effects of CRISPRa perturbation. **A** and **B** UMAPs of T cells colored according to the original study [25] and the $\Phi$-Space-derived CD4 scores (**A**) or activation scores (**B**). **C** and **D** Boxplots showing the effects of individual gene perturbations on T cell activation according to the original activation scores [25] (**C**) and $\Phi$-Space-derived activation scores (**D**). Perturbed genes were categorized according to prior knowledge as positive, negative regulators, where “control” refers to unperturbed cells. Significance levels (Bonferroni-corrected) of each perturbed gene compared to unperturbed cells are indicated (***: $p < 0.001$; **: $p < 0.01$; *: $p < 0.05$)

CD4 and CD8 are two major T cell types. Schmidt et al. [25] claimed that their dataset showed relatively even distributions of CD4 and CD8 T cells. They demonstrated this by defining a CD4 score based on the relative expression levels of CD4 vs CD8 marker genes (Fig. 4A). The $\Phi$-Space phenotype embedding allowed us to define a reference-based CD4 score, measuring how each cell scored on CD4 subtypes relative to CD8 subtypes (see Additional file 2: Section S2). This $\Phi$-Space-derived CD4 score resulted in distributional patterns consistent with the authors (Fig. 4A). Furthermore, to quantify T cell activation, Schmidt et al. defined an activation score based on known gene markers (Fig. 4B). In comparison, our $\Phi$-Space-derived activation score, defined by adding up each cell’s $\Phi$-Space scores of activated T cell subtypes, showed more nuanced stratification of differentially activated T cell states (see Additional file 2: Section S2).

#### $\Phi$-Space enables more interpretable quantification of perturbation effects

Using their activation score, Schmidt et al. [25] found that most of the positive (negative) regulators, i.e., genes known to have positive (negative) effects on T cell activation, resulted in significantly higher (lower) activation scores compared to unperturbed cells (“Control”), as illustrated in Fig. 4C. Notably, the effect of a negative regulator IKZF3 was found indistinguishable from unperturbed cells. In contrast, according to $\Phi$-Space-derived activation score, we found that IKZF3 had a significantly negative effect on activation, consistent with prior knowledge (see Fig. 4D). In addition, more positive regulators were found significant according to $\Phi$-Space. These results have demonstrated that the $\Phi$-Space-derived activation score is biologically more interpretable compared to the activation-marker-based score of the authors.

The rich cell state information contained in our phenotype space embedding also enables the quantification of perturbation effects on T cell states other than activation. For example, we found that enhancing genes TBX21 and GATA3 significantly contributed to T helper 1 (Th1) and T helper 2 (Th2) cell states, respectively (Additional file 1: Fig. S3). These two genes are known to play key roles in Th1 and Th2 differentiations [34, 35].

#### Summary

Through the Perturb-seq study, we illustrated how $\Phi$-Space leveraged the fine immune cell state annotation in a bulk atlas to quantify the effect size of genetic perturbation in a large scale Perturb-seq study. Our results not only provided more interpretable effect size quantification than marker-gene-based approach, but also opened the possibility for more comprehensive evaluations of perturbation effects on T cell states.

### Single-cell multi-omics integration in phenotype space

With the maturation of single-cell multi-omic sequencing, single-cell reference atlases consisting of multimodal datasets, e.g., CITE-seq atlases, are becoming more common [27, 29, 36]. Since both the RNA and the ADT modalities of CITE-seq are useful in characterizing the cell identities related to immune responses, several methods focusing on integrating these two modalities have been proposed [37–39]. However, a method for jointly querying multimodal data against multimodal references is still lacking. We provide a solution to this problem based on the $\Phi$-Space multi-reference annotation (described in Methods “Downstream analyses” section) as follows. First, we trained a PLS model using the RNA features for predicting both COVID-19 severity and 51 fine immune cell types. Then, we trained another PLS model using the ADT features only for predicting the severity and 18 broad cell types, since the number of ADT features (192) is under-powered for predicting the large number of fine cell types. We then concatenated the predicted phenotype scores for the query cells derived using their RNA and ADT features. Our phenotype space analysis provided some fresh insights to the complex interactions between disease conditions and cell type compositions.

#### Phenotype space embeddings provide biology-preserving integration across batches and modalities

**Fig. 5:**
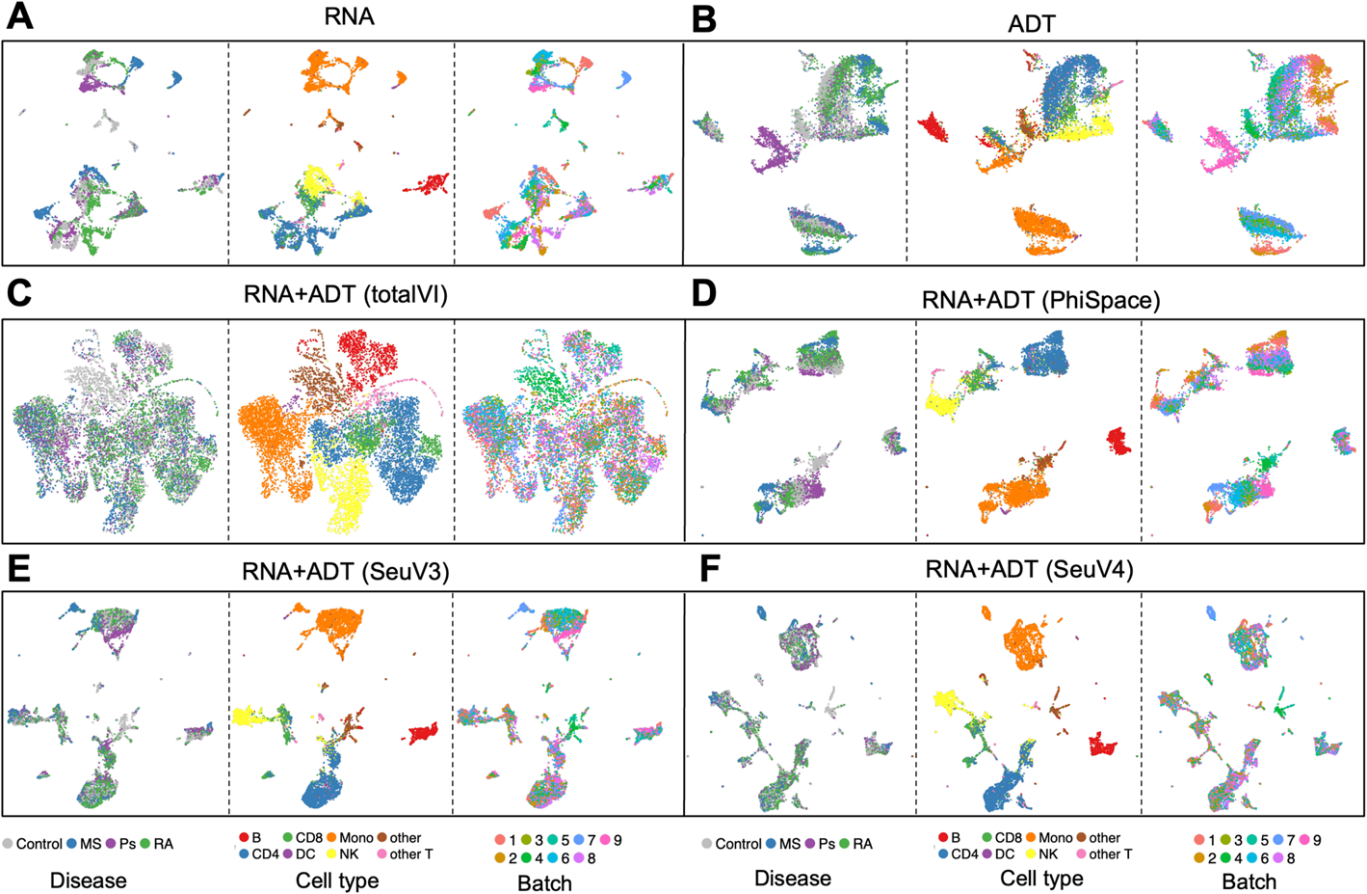
Different representations of query cells in the CITE-seq case study. Uniform manifold approximation and projection plots (UMAPs) based on: **A** and **B** PCs of RNA and ADT features, **C** 64 latent variables from totalVI [37], computed using both RNA and ADT features, **D** 64 PCs of the 87-dimensional $\Phi$-Space phenotype space embedding, computed using both RNA and ADT modalities, and **E** and **F** 64 PCs of the 87-dimensional Seurat V3 and Seurat V4 phenotype space embedding, computed using both RNA and ADT modalities. We obtained the totalVI UMAP results from [28], who set the number of latent variables to be 64. To make our results comparable to the totalVI results, we reduced the dimension of the phenotype space embedding to 64 by PCA. UMAPs of the RNA and ADT modalities showed significant batch effects. TotalVI completely removed the batch effects, but also removed the difference between disease conditions. In contrast, the phenotype space embeddings obtained by $\Phi$-Space, Seurat V3, and Seurat V4 achieved a better balance between removing batch effects and retaining the difference between disease conditions

Both the RNA and ADT modalities of the query data showed significant batch effects (Fig. 5A, B). Barmada et al. [28] used totalVI [37], a state-of-the-art integration method for CITE-seq based on variational autoencoders, to integrate the two modalities while removing batch effects. However, this complete removal of batch effects also blurred the boundaries between cell types and rendered cells from different disease conditions no longer distinguishable (Fig. 5C). In contrast, we computed three versions of phenotype space embeddings from $\Phi$-Space, Seurat V3, and Seurat V4 (Fig. 5D–F, respectively); see Additional file 2: Section S2.1 for how we applied Seurat V3 and V4 [9, 38]. We applied Seurat V3 and V4, rather than SingleR, since SingleR is computationally intensive for large number of cells (see Table 1). Both figures showed well-separated cell types while preserving the differences between disease types. Notably, no batch effects removal step was needed to achieve this separation. This result illustrated the superiority of the phenotype space embedding in preserving complex phenotypic variations and integrating query modalities in the phenotype space.

#### $\Phi$-Space characterizes complex interactions between cell types and disease conditions

**Fig. 6:**
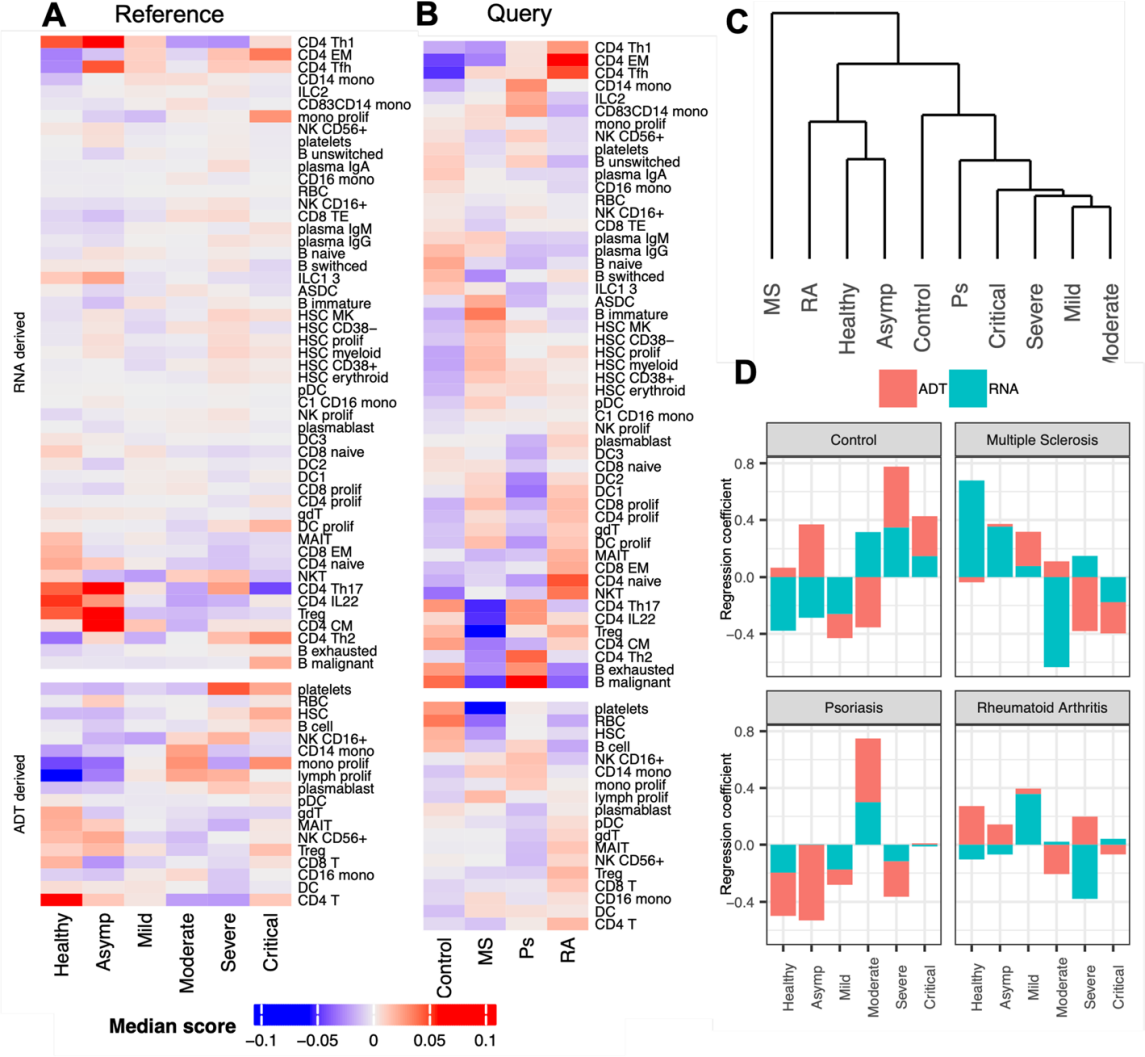
Phenotype space marker selection for CITE-seq data. **A** and **B** Median $\Phi$-Space cell type scores under different disease conditions: **A** scores of reference cells, where columns are COVID-19 severity (Asymp: asymptomatic); **B** scores of query cells, where columns are types of pre-existing autoimmune diseases (Control: severe COVID-19 patients without pre-existing autoimmune diseases; MS: multiple sclerosis; Ps: psoriasis; RA: rheumatoid arthritis). **C** Hierarchical clustering of disease conditions in **A** according to their correlation with cell types. **D** PLS regression coefficients of COVID-19 severity for predicting autoimmune disease types, where red (or teal) bar corresponds to ADT (or RNA) derived scores. We observed a heterogeneity of COVID-19 patients with different types of pre-existing autoimmune diseases, in terms of cell type composition and disease severity

Our phenotype space approach of multi-omics integration also provides a new way of quantifying the interaction between cell types and biological conditions (e.g., disease conditions, age groups). Conventional analyses typically first annotate the single cells, then calculate cell type proportions under each biological condition [27, 40, 41]. However, this approach is very sensitive to the way cell types are defined, which hinders the direct quantitative comparison of cell type compositions derived in different studies. With $\Phi$-Space, we solve this problem by analyzing both the reference and query disease conditions in the same phenotype space, so that cell type compositions are directly comparable. The $\Phi$-Space predicted cell type scores differentiated the enrichment or depletion of cell types according to disease conditions (Fig. 6A and B), in agreement with [27, 28] (discussed below). Thus, we showed that with $\Phi$-Space we can compare cell type compositions across biological conditions and studies. Of note, we could use Seurat V3 and V4 scores to plot similar heatmaps (Additional file 1: Fig. S5). However, the plots were not interpretable due to the overly high proportion of zeros in the scores.

Based on the ADT-derived results in severe COVID-19 patients compared to healthy donors, we observed an increased presence of B cells, platelets, plasmablasts, hematopoietic stem cells (HSCs), proliferating monocytes, and proliferating lymphocytes (Fig. 6A). In addition, based on the RNA derived scores, we observed the enrichment of type 1 helper T cells (CD4 Th1), IL22 expressing CD4 T cells (CD4 IL22), and regulatory T cells (Treg) in asymptomatic patients. Both observations are consistent with findings in [27]. Moreover, consistent with [28], we observed in the query data (Fig. 6B) an overall lack of phenotypic signatures characterizing severity, such as HSCs, platelets, and plasmablasts, in COVID-19 patients with autoimmune diseases, compared to the severe COVID-19 patients in the control group. Furthermore, [28] identified an enrichment of type 2 helper T cells (Th2) in psoriasis (Ps) patients and the enrichment of CD4 memory T cells in rheumatoid arthritis (RA) patients, which were confirmed in Fig. 6B.

In addition to these known findings, our $\Phi$-Space analysis provided a much more nuanced characterization of the heterogeneity of autoimmune disease types. For example, compared to psoriasis (Ps), both rheumatoid arthritis (RA) and multiple sclerosis (MS) patients tended to display stronger mature DC (DC1, DC2, and DC3) presence, a feature characterizing healthy donors (Fig. 6A; see also [27]). In addition, Ps patients were characterized by an enrichment of the exhausted and malignant B cells, which were both indicators for greater COVID-19 severity [27]. These additional findings illustrated the heterogeneity of the immune landscape of COVID-19 patients with different types of autoimmune diseases. This heterogeneity was further illustrated by a hierarchical clustering analysis of cell type composition between disease conditions in Fig. 6C. In light of the relatively small sample size involved in [28], more samples from COVID-19 patients with autoimmune diseases need to be collected for a thorough investigation of this heterogeneity.

#### $\Phi$-Space highlights disease severity markers of autoimmune conditions

To directly characterize disease severity of COVID-19 patients with autoimmune disease, we conducted a phenotype space marker selection. We used the query cells’ $\Phi$-Space embeddings to predict their autoimmune disease types (Fig. 6D). This allowed us to directly assess how cell type enrichment and COVID-19 severity discriminate autoimmune disease types (only COVID-19 severity was visualized). As expected, the phenotypic variation of the control group consisting of severe COVID-19 patients without any autoimmune disease were best predicted by severe and critical COVID-19 conditions. In contrast, MS and RA were better predicted by healthy, asymptomatic, and mild conditions. Finally, Ps was better predicted by the moderate condition. These findings were consistent with our findings shown on Fig. 6A–C. These figures illustrated from different angles the heterogeneity of the immune landscapes of COVID-19 patients with different pre-existing autoimmune diseases.

#### Summary

Through the CITE-seq case study, we demonstrated how a simple concatenation of phenotype space embeddings derived using different omics modalities leads to integrative analyses of complex interactions of cell phenotypes. As a generic modeling strategy, our multi-omics annotation can also be applied using soft classification methods other than PLS, such as Seurat V3 and V4. Compared to a direct integration of the omics features, our phenotype space approach achieved a better balance between removing batch effects and preserving difference between disease conditions. This enables us to conduct insightful phenotype space marker analyses, where we could directly compare cell type compositions and disease conditions from different studies. In particular, our approach provides a fully quantitative alternative to the largely qualitative approach used in [28].

### Flexible cell type transfer within and across omics

So far, we have described the ability of $\Phi$-Space to perform soft classification. Here we show that $\Phi$-Space also provides high quality hard classification for both within- and across-omics cell type transfer. After within- or across-omics annotation of the query data, we discretized query cells’ phenotype space embeddings to obtain their predicted cell type labels (see Methods section). We designed two cross-validation (CV) procedures to benchmark the accuracy of $\Phi$-Space against several state-of-art methods in both within- and across-omics label transfer (see summary of case studies in [Sec Sec3] section). We considered the complex batch effects in the dataset when designing these CV procedures: they mimic the realistic scenario where the reference and query datasets are generated under different experimental conditions [6, 10]. The balanced classification errors were then calculated based on the ground truth cell type labels (Additional file 2: Section S2.2).

#### $\Phi$-Space accurately transfers cell type labels between scRNA-seq datasets

**Fig. 7:**
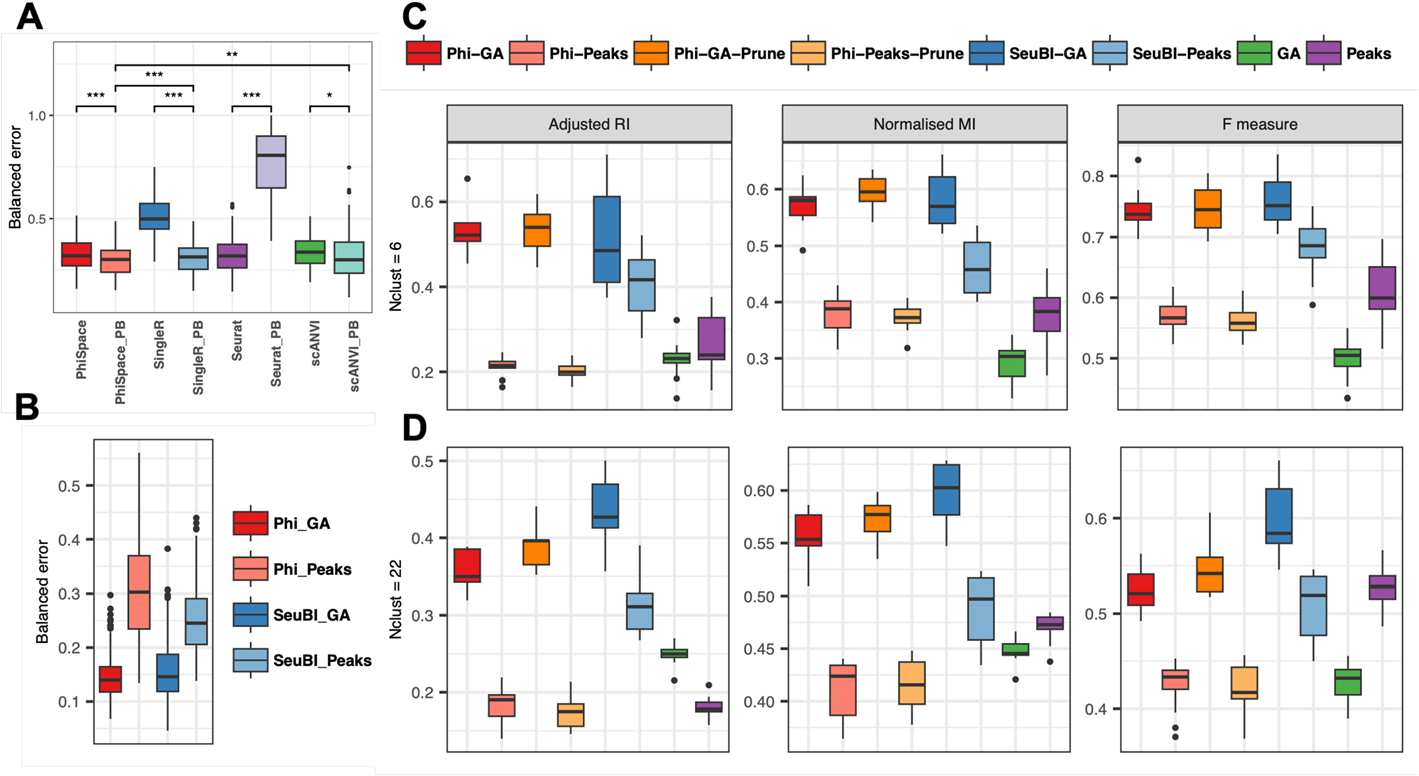
Benchmarking hard classification and phenotype space clustering performances. **A** and **B** Balanced classification errors for: **A** within-omics annotation, where the scRNA-seq query was annotated according to $\Phi$-Space, SingleR, Seurat V3, and scANVI using scRNA-seq references or their pseudo-bulk versions, “PB”; **B** cross-omics annotation, where the scATAC-seq query was annotated according to $\Phi$-Space (Phi) and Seurat bridge integration (SeuBI) using scRNA-seq reference and bimodal bridge. “Peaks” and “GA” indicate ATAC measurements represented as either peaks or gene activity (GA) scores. **C** and **D** Three metrics (adjusted Rand index, normalized mutual information, and F measure) for evaluating k-means clustering based on phenotype space embeddings generated by different methods, or on PCs of either GA scores or peaks. We also applied score pruning to $\Phi$-Space scores (i.e., deleting negative scores), which result in their “-Prune” versions. The number of k-means clusters were set to **C** 6—the number of ground truth major cell types; or **D** 22—the number of fine cell types. $\Phi$-Space performance was comparable to state-of-the-art methods in both within- and cross-omics annotation. In terms of clustering, $\Phi$-Space was always among the top two best performing methods. Score pruning (e.g., Phi-GA-Prune) tended to improve the performance of $\Phi$-Space

For within-omics annotation, $\Phi$-Space led to similar classification errors compared to the state-of-the-art Seurat V3 and scANVI, while SingleR did not perform well. Then using pseudo-bulk references, the performance of $\Phi$-Space was similar to SingleR and scANVI, while Seurat underperformed (Fig. 7A). In particular, $\Phi$-Space, SingleR, and scANVI all benefited from a pseudo-bulk approach as opposed to Seurat V3.

#### $\Phi$-Space accurately transfers cell type labels from scRNA-seq to scATAC-seq datasets

In our second CV procedure, we used both the RNA and ATAC modalities of the 10x multiome dataset to evaluate cross-omics label transfer performances of $\Phi$-Space and Seurat bridge integration (SeuBI) [16]. ATAC measurements can be represented as either peaks or gene activity (GA) scores. Therefore, we evaluated the classification performance of $\Phi$-Space and SeuBI on each of these representations. On GA scores, both $\Phi$-Space and SeuBI had similar classification error rates, with the $\Phi$-Space errors being slightly less variable (Fig. 7B); on peaks, the classification error rates were noticeably higher than on the GA scores for both methods, suggesting that GA scores rather than peaks lead to better performance for cell type transfer.

#### Phenotype space clustering outperforms omics space clustering

A limitation of fully supervised cell type transfer is that cell types present in the query are not necessarily defined in the reference (e.g., cancer cells that are not present in healthy reference). To solve this problem, we can use unsupervised clustering to identify homogeneous clusters of cells, and then identify the cell type of each cell cluster based on its marker genes. However, fully unsupervised clustering does not make use of any reference even when available. Our phenotype space clustering strategy, i.e., clustering based on the phenotype space embedding of cells (described in Additional file 2: Section S1), combines the strengths of supervised and unsupervised cell type identification.

We adapted the above CV framework for evaluating clustering, where for each CV iteration we simply stacked the phenotype space embeddings of different batches of query cells (from either $\Phi$-Space or SeuBI) and applied k-means clustering; we also applied k-means to pruned $\Phi$-Space phenotype space embeddings (Additional file 2: Section S2.3). As a baseline, we applied k-means to peaks and GA scores directly to conduct fully unsupervised clustering (Additional file 2: Section S2.3). We evaluated all methods’ ability to recover either the 6 broad ground truth cell types or the 22 fine ground truth cell types or 22 respectively in each k-means analysis (by specifying $k = 6$ or $k=22$ in k-means), and used three commonly used metrics to evaluate the clustering results (described in Additional file 2: Section S2.4; higher metrics suggest better clustering).

$\Phi$-Space and SeuBI were the best performing methods, according to all three metrics, when using the GA scores to recover broad cell types (Fig. 7C). However, the performances of SeuBI on either GA or peaks were much more variable compared to $\Phi$-Space on GA scores, indicating that $\Phi$-Space might be more robust against the quality of reference and bridge datasets. Moreover, pruning tended to improve the performance of $\Phi$-Space clustering in most cases. SeuBI and the $\Phi$-Space with pruning were the best performing method in recovering fine cell types when applied to GA scores (Fig. 7D). Overall, $\Phi$-Space and SeuBI always performed better on the GA scores than the peaks, as observed in the classification analyses (Fig. 7B). This was likely due to the higher signal-to-noise ratio of GA scores compared to peaks. Indeed, since individual peaks corresponded to sub-gene regions, most peaks were not open in the scATAC-seq data. This made the peak-by-cell matrix much sparser compared to the gene-by-cell matrix. By aggregating peaks to genes, we effectively reduced the sparsity and enhanced the signal in scATAC-seq pertaining to cell type identification. It seemed that $\Phi$-Space benefited more from this reduction of sparsity compared to SeuBI, as demonstrated by the much larger difference in the performance of Phi-GA and Phi-Peaks compared to the SeuBI counterparts.

#### Summary

Through the scATAC-seq case study we showed that $\Phi$-Space led to good performances in both within- and cross-omics cell type transfer, comparable to several state-of-the-art methods. Of note, our application of SeuBI on GA scores is novel, as SeuBI was originally designed for utilizing peaks [16]. Our benchmarking results suggested that using GA scores (aggregated peaks) might be beneficial for cell type transfer tasks, due to the their higher signal-to-noise ratio compared to peaks.

## Discussion

We developed $\Phi$-Space to address numerous challenges faced by state-of-the-art automated annotation methods, including identifying continuous cell states, dealing with batch effects in reference, utilizing bulk references, and including multi-omic references and queries. $\Phi$-Space uses soft classification to phenotype cells on a continuum. We then use the continuous annotation, or phenotype space embedding, to reduce the dimensionality of our data for various downstream analyses.

Through the four biological case studies (DC, Perturb-seq, CITE-seq, and scATAC-seq), we have demonstrated the versatile use of $\Phi$-Space in continuous phenotyping. While our case studies featured some alternative methods, none of these methods can be applied to all modeling tasks described above. In contrast, thanks to the flexibility of PLS regression and our phenotype space modeling strategies, $\Phi$-Space can be applied to a much wider range of tasks, as summarized below.

First, $\Phi$-Space can characterize developing cell states. In the DC case study, $\Phi$-Space characterized developing cell states of the induced dendritic cells, which were not defined in the reference. In the scATAC-seq case study, via the clustering analysis we showed that $\Phi$-Space better preserved cell types defined in query datasets. This greatly extends the utility of automated cell annotation beyond hard cell type classification.

Second, $\Phi$-Space is robust against batch effects in both reference and query datasets, without requiring additional batch effects correction. This was demonstrated in all three case studies. This greatly simplifies the cell annotation workflow, leading to reduced computational cost.

Third, $\Phi$-Space is flexible enough to be extended to annotation tasks involving multiple omics types. In addition to within-omics annotation (DC and Perturb-seq cases), $\Phi$-Space can also accomplish cross-omics annotation (scATAC-seq case) and multi-omics annotation (CITE-seq case). In particular, our cross-omics annotation approach cannot be replicated by alternative methods such as SingleR, Seurat V3, or Seurat V4 as we use the bridge dataset to annotate the query, then train a PLS regression model to predict a continuous phenotype matrix—the latter cannot be achieved by these alternative soft classification methods. As single-cell multi-omics data are becoming increasingly common [42], we anticipate that cross-omics and multi-omics annotation will be increasingly useful in biological research.

Fourth, $\Phi$-Space overcomes strong technical differences between reference and query sequencing platforms. In the DC case study, we used bulk reference to annotate scRNA-seq query, utilizing a large number of bulk studies. In the scATAC-seq case study, we used scRNA-seq reference to annotate scATAC-seq query. On the one hand, this approach recycles high-quality data from conventional sequencing technologies. On the other hand, emerging sequencing platforms (e.g., the fast evolving spatial transcriptomics [43]) have very different characteristics compared to well established platforms, and we anticipate that $\Phi$-Space will help transfer biological information to these newly generated datasets.

Methodologically speaking, the essence of $\Phi$-Space, namely the phenotype space analysis, is the use of soft classification as dimension reduction. This modeling strategy is greatly under-utilized in computational biology. Our work proved that this modeling strategy leads to very flexible tools for modeling complex phenotypic variations in multi-omics data. In addition, $\Phi$-Space used a particular soft classification method, PLS, a workhorse method deployed in routine analyses of many scientific disciplines [44–47]. However, any soft classification method can be used for $\Phi$-Space type of analyses. This further proves the value of $\Phi$-Space, not just as a versatile computational toolkit for continuously modeling cell phenotypes, but also as a generic modeling strategy.

$\Phi$-Space also has great potential in uncovering complex interactions of different cell phenotypes. This was demonstrated in the CITE-seq case study, where we profiled cellular compositions of COVID-19 patients. Despite a considerable amount of existing work, it remains an open problem how pre-existing autoimmune diseases contribute to COVID-19 severity [48–51]. This is partly due to the complex interaction between the two layers of phenotypic variations, cell types and the donors’ disease conditions. In addition, the query data in the CITE-seq case study from [28] were particularly challenging to analyze since the disease condition is seriously confounded with experimental batches. Both challenges were effectively dealt with by $\Phi$-Space.

$\Phi$-Space’s flexibility opens many possible future research directions. One limitation of the current work is that we did not make use of bulk multi-omic reference atlases. A potential extension of $\Phi$-Space is the integration of bulk and single-cell multi-omics data. The motivation is that most existing single-cell multi-omics technologies only generate data with two omics types [42]. Hence, the abundance of bulk datasets with 3 or more omics types (e.g., TCGA) may mitigate the lack of such single-cell datasets. Based on some preliminary results, we found it promising to apply $\Phi$-Space to deconvolute cell types in bulk samples from solid tissues (e.g., breast tumor) based on scRNA-seq references. Another possible extension is to develop $\Phi$-Space into a unified approach for annotating spatial transcriptomics data generated by radically different platforms.

## Conclusions

In conclusion, we have presented a powerful solution to address the challenge of identifying complex cell states in single-cell multi-omics data. We illustrated the flexibility and robustness of $\Phi$-Space by four biological case studies, involving bulk RNA-seq, scRNA-seq, Perturb-seq, CITE-seq, and scATAC-seq data. We anticipate that the ability of $\Phi$-Space to uncover continuous phenotypic information will facilitate the discovery of spatial phenotypic patterns. We believe this toolkit will empower biological research such as developmental and cancer biology, which feature complex and atypical cell identities that are hard to identify using conventional methods.

## Methods

### $\Phi$-Space continuous phenotyping

In the following sections, we denote the reference dataset by $(X_{\text {ref}}, Y_{\text {ref}})$, where $X_{\text {ref}}$ is a $(N\times P)$ omics feature matrix of normalized values, with *P* omics variables measured on *N* samples (either bulk or single-cell; depicted in Fig. 1A). The phenotype matrix $Y_{\text {ref}}$ is a $(N\times K)$ dummy matrix that represents the *K* phenotype labels of the *N* samples, that is, each column of $Y_{\text {ref}}$ represents a phenotype label (e.g., a cell type or sample source) and a sample is assigned the value 1 if it has that label or $-1$ otherwise. Importantly, we allow each sample to have more than one label (i.e., a sample can be both dendritic cell and in vitro), thus each row of the matrix *Y* may contain multiple values of 1.

**Training step.** To model the relationship between the omics feature matrix $X_{\text {ref}}$ and the multivariate $Y_{\text {ref}}$, we apply partial least squares regression (PLS) [21, 47] to estimate a low-rank regression coefficient matrix *B* of size $(P\times K)$ so that $Y_{\text {ref}} \approx X_{\text {ref}} B$ (depicted in Fig. 1B). The rank of *B* is equal to the number of PLS components, by default set to *K*, the number of all phenotype labels. Additional file 2: Section S1 describes in detail how to optimize the selection of this parameter.

Feature selection is often desired to remove unwanted variation contained in noisy features and to increase the model’s interpretability. PLS regression allows for feature selection in $X_{\text {ref}}$ so that only the top features that are highly predictive are selected in PLS; see Additional file 2: Section S1 for details.

Since $\Phi$-Space only had the two tunable parameters list above, it is possible to use a fully data-driven approach to select them, such as the cross-validation procedure introduced in Additional file 2: Section S1. In addition, we observed that in practice the selection of the number of PLS components (i.e., the rank of *B*) had larger effects on model performance compared to the number of features. The feature selection module of $\Phi$-Space is optional and is mainly for increasing interpretability. In contrast, other baseline methods such as Seurat V3 [9], Seurat V4 [38], and scANVI [6] have much larger numbers of tunable parameters. For example, the “FindTransferAnchors” function in Seurat V3 alone has tunable parameters such as “dims,” “k.anchor,” and “k.score.” Having multiple tunable parameters makes it difficult for these methods to adopt a fully data-driven approach for parameter tuning due to high computational costs.

**Annotation step.** To transfer the phenotypic information learnt from the reference, we consider the three analytical tasks:

1. **Within-omics annotation** (Fig. 1B). When the query data $X_{\text {query}}$ of size $(M\times P)$, with *M* denoting the number of query cells, contain the same omics variables as $X_{\text {ref}}$, we compute the annotation denoted $\hat{Y}_{\text {query}}$ based on $X_{\text {query}}$ and the regression coefficient matrix *B* from PLS($X_{\text {ref}}, Y_{\text {ref}}$), as detailed in Additional file 2: Section S1. We illustrate this analysis in DC and Perturb-seq case studies in Continuous characterization of developing cell states and Quantifying effect sizes of genetic perturbations in Perturb-seq sections.
2. **Multi-omics annotation** (Fig. 1C). When both the reference $(X_{\text {ref}}, Z_{\text {ref}})$ and the query $(X_{\text {query}}, Z_{\text {query}})$ contain two modalities, we train PLS($X_{\text {ref}}, Y_{\text {ref}}$) and PLS($Z_{\text {ref}}, Y_{\text {ref}}$), and then concatenate the predicted scores to obtain the annotation $\hat{Y}_{\text {query}}$. We illustrate this analysis in the CITE-seq case study in Single-cell multi-omics integration in phenotype space section.
3. **Cross-omics annotation** (Fig. 1D). When the query data $Z_{\text {query}}$ of size $(M\times Q)$, with *M* denoting the number of query cells and *Q* the number of omics variables, is of a different omics type compared to the reference, we use a bimodal bridge dataset $(X_{\text {bridge}}, Z_{\text {bridge}})$ similar to the approach of [16]. The bimodal bridge shares omics features with both the reference and the query as an intermediate. To achieve this, we first compute the annotation $\hat{Y}_{\text {bridge}}$ based on $X_{\text {bridge}}$ and the regression coefficient matrix *B* from PLS($X_{\text {ref}}, Y_{\text {ref}}$) (see point 1 above). We then train a second PLS model PLS($Z_{\text {bridge}}, \hat{Y}_{\text {bridge}}$) to obtain the second regression coefficient matrix $B_{\text {bridge}}$ of size $(Q\times K)$. The predicted phenotype of the query $\hat{Y}_{\text {query}}$ is then calculated based on $Z_{\text {query}}$ and $B_{\text {bridge}}$. We illustrate this analysis in the scATAC-seq case study in Flexible cell type transfer within and across omics section.

In all cases above, the main output of $\Phi$-Space is the annotation of the *M* query cells on a continuous scale, denoted $\hat{Y}_{\text {query}}$ of size $(M\times K)$, which is calculated either based on $X_{\text {query}}$ (within-omics), $Z_{\text {query}}$ (cross-omics), or both $X_{\text {query}}$ and $Z_{\text {query}}$ (multi-omics). We refer to $\hat{Y}_{\text {query}}$ as the *phenotype space embedding* of the query data.

### Downstream analyses

The phenotype space embedding $\hat{Y}_{\text {query}}$ contains rich phenotypic information, which we then leverage in the following downstream analyses.

*Phenotype space reference mapping* (Fig.1E). Mapping the query data to a reduced-dimensional space of the reference is a common way for visualizing and comparing the reference and query samples in the same low-dimensional space. However, due to the platform effects between reference and query, reference mapping based on omics features is not always straightforward [14, 15, 52]. This problem can be solved by doing reference mapping based on the phenotype space embeddings of reference and query samples. To achieve this, once we have computed $\hat{Y}_{\text {ref}}$ from the reference (Fig. 1B), we obtain the principal components (PCs) of $\hat{Y}_{\text {ref}}$ and then map $\hat{Y}_{\text {query}}$ to these PCs (Fig. 1E). In the DC case study (Continuous characterization of developing cell states section), we will demonstrate that our phenotype space reference mapping effectively overcomes the discrepancy between the reference and the query datasets. This discrepancy can be caused by different sequencing platforms (e.g., bulk vs single-cell) or even different omics types (e.g., scRNA-seq vs scATAC-seq).

*Phenotype space marker selection* (Fig. 1F). Conventional marker selection identifies omics features, such as genes, that distinguish particular groups of samples. Analogously, given the phenotype space embedding $\hat{Y}_{\text {query}}$ and some known grouping of the query cells (e.g., donor’s disease condition), we can identify phenotypic markers of different groups of query cells. In the CITE-seq case study (Single-cell multi-omics integration in phenotype space section), we show how we can identify cell type and disease severity signatures of COVID-19 patients with different pre-existing autoimmune conditions.

*Phenotype space clustering *(Fig. 1G). The phenotype space embedding $\hat{Y}_{\text {query}}$ of the query data $X_{\text {query}}$ (resp. $Z_{\text {query}}$ for cross-omics annotation) is robust against possible batch effects in $X_{\text {query}}$ (resp. $Z_{\text {query}}$), as we demonstrate in the CITE-seq and scATAC-seq case studies (Single-cell multi-omics integration in phenotype space and Flexible cell type transfer within and across omics sections). This is because $\hat{Y}_{\text {query}}$ is derived from the PLS regression model learned from the reference rather than the query, and hence is agnostic of the batch effects in the query omics space. This means that $\hat{Y}_{\text {query}}$ can be interpreted as a dimension reduction technique that preserves biological sources of variation, and is suitable for identifying biologically meaningful clusters of cells. A similar approach has been used by [12] who identified a novel subtype of macrophage in their scRNA-seq data. Our phenotype space clustering is a generalization of their approach to cluster other omics types. See Flexible cell type transfer within and across omics section for an extension to scATAC-seq data.

*Hard classification* (Fig. 1H). Similar to conventional cell type annotation methods such as Seurat V3 [9] and SingleR [12], we can also derive predicted cell type labels for query cells. To achieve this, we assign to each query cell the cell type label with the highest predicted scores in each row of $\hat{Y}_{\text {query}}$, akin to hard classification. We demonstrate in our benchmark studies that $\Phi$-Space is able to achieve accurate cell type annotation in the scATAC-seq case study (Flexible cell type transfer within and across omics section).

### Additional notes

*Unequal number of features in reference and query.* It is often the case that the reference and the query datasets contain different number of features (e.g., genes). Unless otherwise stated, the number of features in this paper always refer to the number of common features shared by the reference and the query.

*Scalability constraints.* The computational complexity of $\Phi$-Space mainly comes from the training step, i.e., PLS regression, which has complexity $O(r\,\text {poly}(PK) + r\,\text {poly}(NP))$, where *r* denotes the number of PLS components, *P* the number of omics features, *K* the total number of phenotypes, and *N* the sample size. Since we usually have $r=K<< \min (N,P)$, the scalability constraints of $\Phi$-Space come from *N* and *P*. In our case studies, we found training $\Phi$-Space much faster than other benchmark methods. For much larger *N* or *P* (e.g., millions of cells), we suggest training $\Phi$-Space on a subset of training data using the “subsample” function in the $\Phi$-Space package [53], which ensures balanced downsampling of all phenotypes.

*Number of cell types in reference.* We recommend, when possible, using a single reference dataset with rich enough cell phenotypes. This type of reference datasets often come from large-scale cell atlases. When such as dataset is hard to find, $\Phi$-Space allows the user to input multiple reference datasets, where together these datasets cover a rich enough range of cell phenotypes. The richness of the reference phenotype space is hard to quantify, but rather has to be judged biologically. For example, in the DC case study, although our target was to validate if the reprogrammed HEFs have gained DC identity, it was helpful to include other transcriptionally similar myeloid cell types to enhance the statistical power. In general, we observed that including more cell types helped improving the performance of $\Phi$-Space.

## Supplementary information

Additional file 1: Supplementary Figures and Tables. Additional figures and tables supporting our biological case studies.

Additional file 2: Supplementary Methods. Additional details of the $\Phi$-Space method; additional details of the analyses in case studies.

## Data Availability

The $\Phi$-Space R package is available on GitHub [53], under the MIT licence. The source code for reproducing results in this paper has been deposited at Zenodo [54]. All data used in this paper are publicly available. The bulk dendritic cell (DC) atlas [23] is available at Stemformatics [55]. The Rosa et al. scRNA-seq dataset of induced DCs [22] is deposited at Gene Expression Omnibus (GEO) under accession number GSE189612 [56]. The Haniffa lab [27] COVID-19 CITE-seq data is deposited at Array Express under accession number E-MTAB-10026 [57]; the Vento-Tormo lab [28] COVID-19 CITE-seq is deposited at European Genome-Phenome Archive (EGA) under accession number EGAD00001007982 [58]; processed versions of both datasets are available at the COVID-19 Cell Atlas [59]. The BMMC 10X Multiome dataset [30] is deposited at GEO under accession number GSE194122 [60]. The bmcite dataset [9] is deposited at GEO under accession number GSE128639 [61]; its processed version can be downloaded using the R package SeuratData [62].
