# Supplementary material for: Φ-Space: continuous phenotyping of single-cell multi-omics data
Source: Genome Biol. 2025 Sep 30;26:323. doi: 10.1186/s13059-025-03755-8 (PMC12482742; doi:10.1186/s13059-025-03755-8)
Supplement: Supplementary file 1 — Additional file 1: Supplementary Figures and Tables. Additional figures and tables supporting our biological case studies. [file 13059_2025_3755_MOESM1_ESM.pdf]

Supplementary Figures and Tables for "Φ-Space: Continuous phenotyping of single-cell multi-omics data"

Jiadong Mao[1], Yidi Deng[1], Kim-Anh Lê Cao[1,*]

[1]Melbourne Integrative Genomics, School of Mathematics and Statistics, The University of Melbourne, Australia

∗ corresponding author: kimanh.lecao@unimelb.edu.au

S1 Supplementary figures

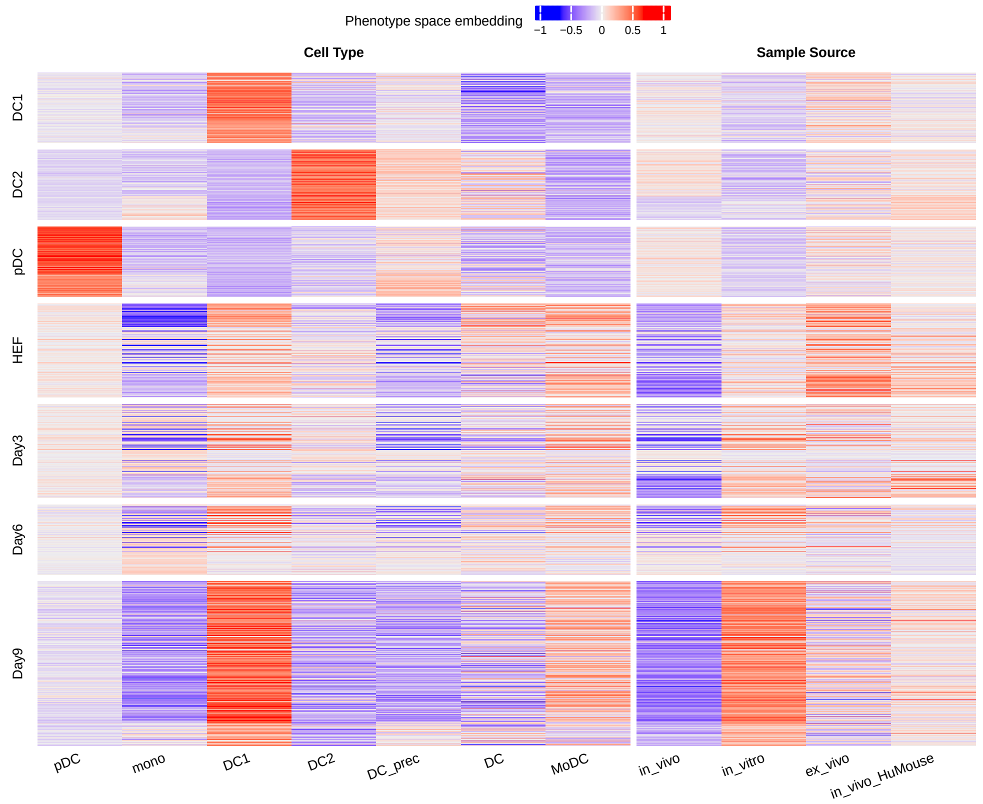

Fig. S1 DC case study Φ-Space output. Heatmap showing phenotype space embeddings of query cells, where each row is a query cell and each column is a phenotype in the bulk reference. Query cell types include DC1: type 1 conventional dendritic cell (DC); DC2: type 2 conventional DC; pDC: plasmacytoid DC; HEF: human embroyonic fibroblast; Day3, Day4, Day9: HEFs after 3, 6 and 9 days of reprogramming towards DC. Reference cell types include, in addition to DC1, DC2 and pDC, mono: monocytes; DC_prec: DC precursor; DC: DC with unknown subtypes; MoDC: monocyte derived DC. The reference sample source in_vivo_HuMouse refers to *in vivo* cells from humanised mice.

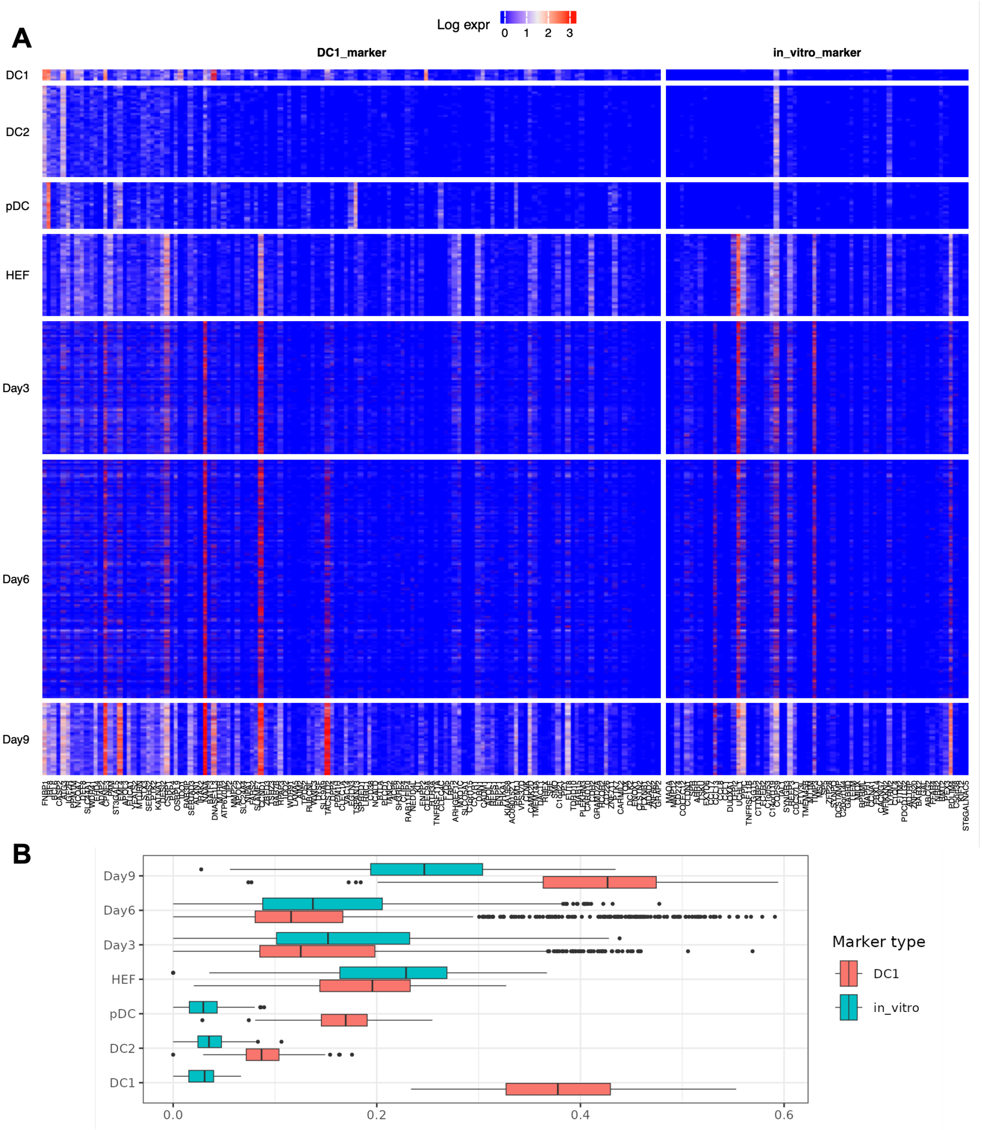

Fig. S2 DC case study marker validation. To further validate the Day 9 reprogrammed human embroyonic fibroblasts (HEFs) gained *in vitro* type 1 conventional dendritic cell (DC1) identity, we did a marker analysis using DC1 and *in vitro* marker genes identified by Elahi et al. [1]. **A** Heatmap showing log-normalised expression levels of marker genes. Day 9 reprogrammed HEFs highly expressed both DC1 and *in vitro* markers. In contrast, the *in vivo* DC1s highly expressed DC1 markers but not *in vitro* markers. **B** Mean expression levels of DC1 and *in vitro* markers in different cell types. We observed that Day 9 reprogrammed HEFs had DC1 marker gene expression levels comparable to *in vivo* DC1s, with significantly higher *in vitro* marker gene expression level scompared to all other cell types.

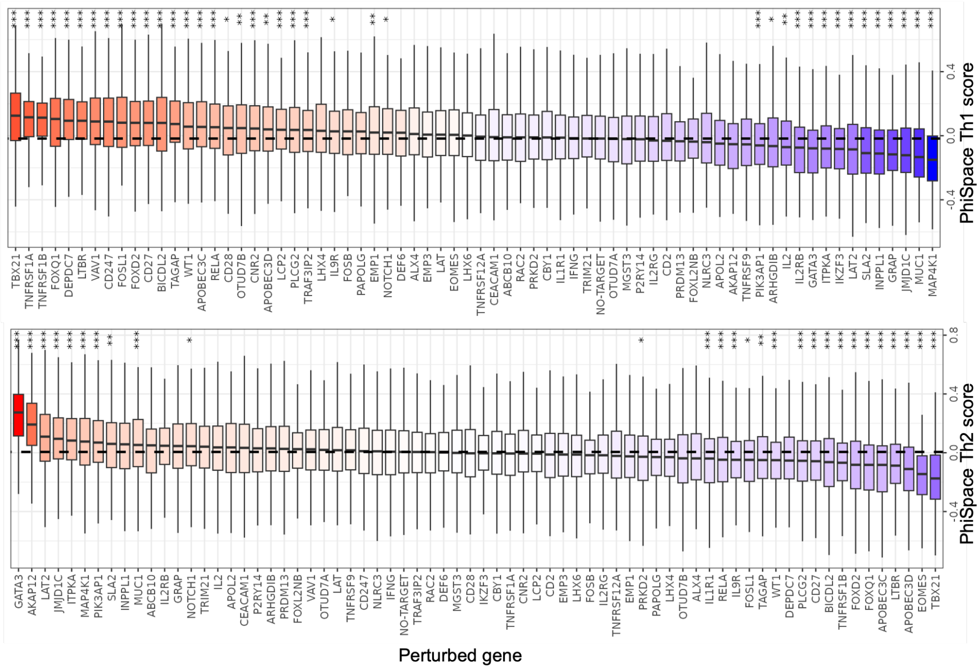

Fig. S3 Quantifying effects of genetic perturbations on T cell differentiation. Φ-Space allows us to quantify how the perturbation of individual genes affected T cell differentiation in the Perturb-seq data of Schmidt et al. [2]. We illustrate this by plotting the boxplots of Φ-Space scores of T helper 1 (Th1) and T helper 2 (Th2) cell types. Number of asterisks represents significance level (Bonferroni-corrected) of each perturbed gene compared to unperturbed cells: ***, $p < 0.001$; **, $p < 0.01$; *, $p < 0.05$.

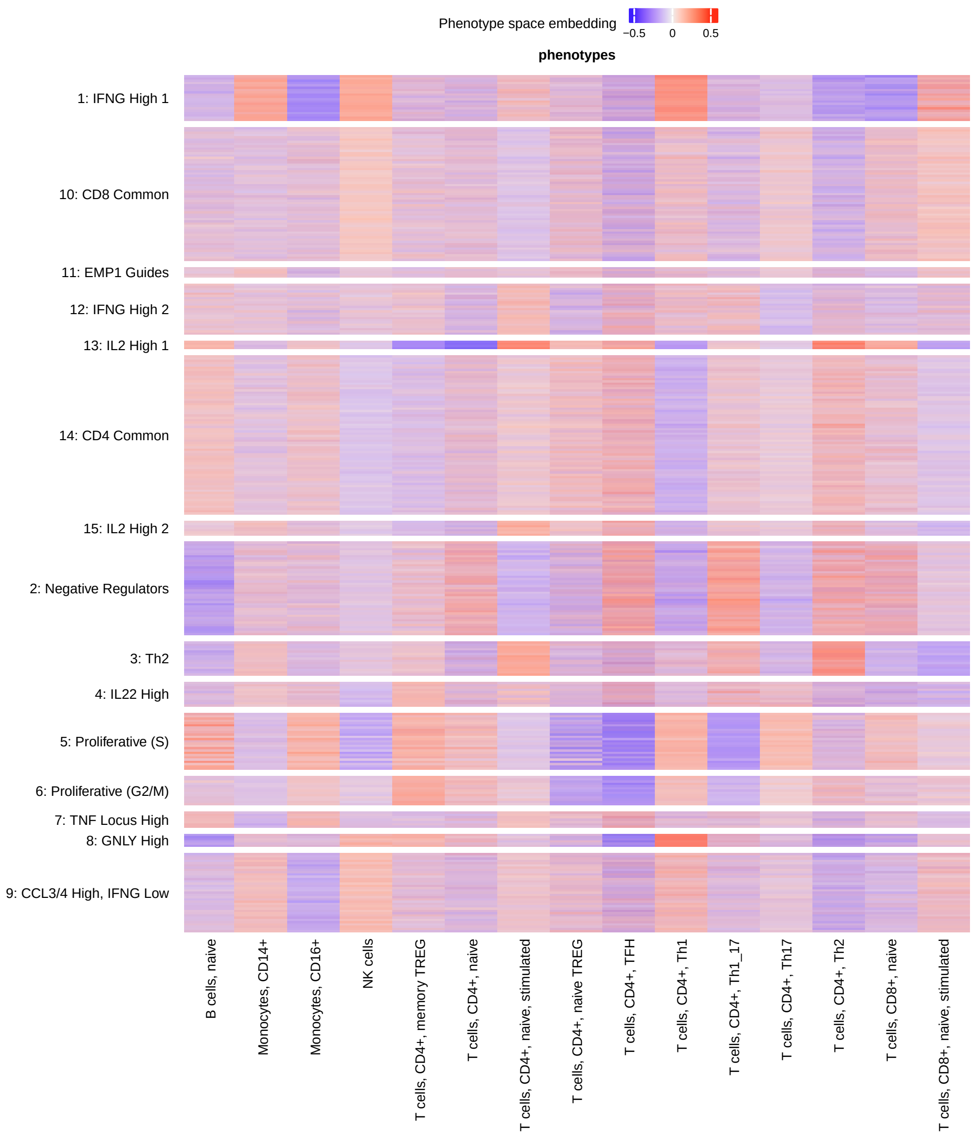

Fig. S4 Phenotype space embedding of cells in Perturb-seq data. Heatmap showing phenotype space embedding of cells in the Perturb-seq data of Schmidt et al. [2]. Row labels are cell cluster labels in Perturb-seq data; column labels are cell types defined in the DICE bulk RNA-seq reference [3].

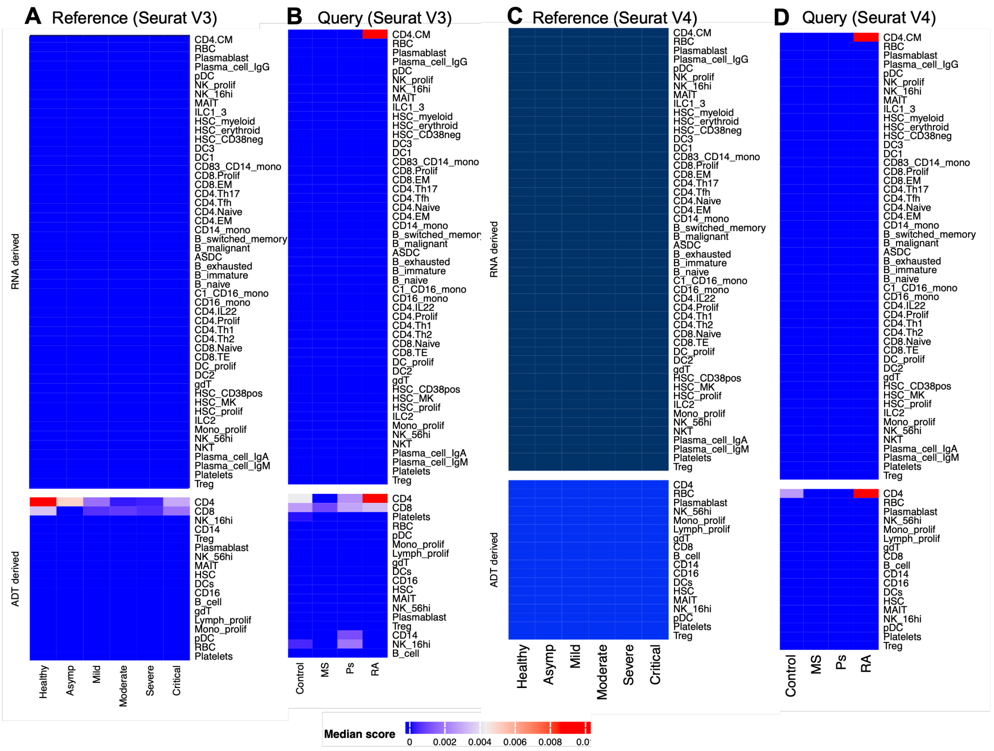

Fig. S5 CITE-seq case study. **A**&**B** (**C**&**D**) are the same as Fig 6A&B except that Seurat V3 (Seurat V4) predicted cell type scores are used. Most median scores are zero due to the high proportions of zeros in the score matrix.

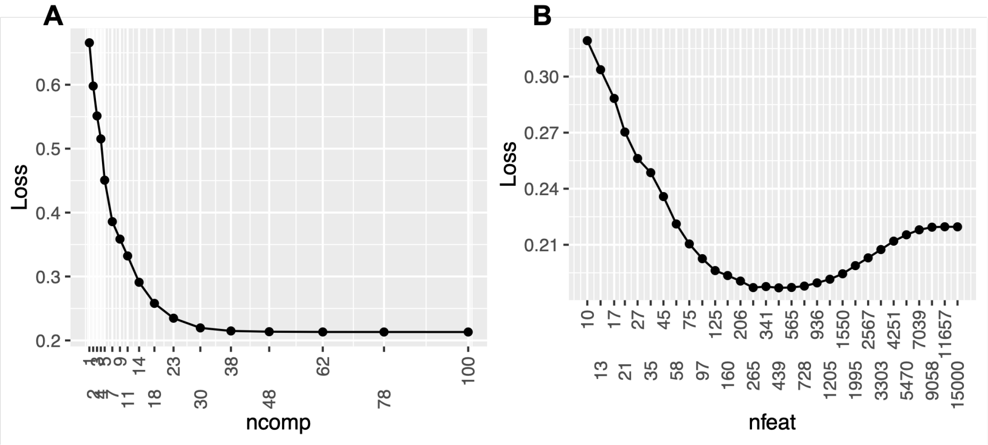

Fig. S6 Φ-Space parameter tuning for PLS components and feature selection. Cross-validated residual sum of squares (RSS) defined in Additional File 2: Section S1 (y-axis) for selecting **A** `ncomp` and **B** `nfeat`. Here we can select `ncomp` = 30 and `nfeat` = 265 since they result in minimal RSS values.

S2 Supplementary tables

Table S1 Merging fine cell types in the bmcite dataset to broad ones. We manually merged the fine cell types defined in the bmcite dataset [4] to broad cell types, for benchmark studies on cross-omics annotation (see Section 2.1).

Fine cell types	Broad cell types
CD14 Mono	Mono/DC
CD16 Mono	Mono/DC
CD4 Memory	T cell
CD4 Naive	T cell
CD56 bright NK	NK
CD8 Effector_1	T cell
CD8 Effector_2	T cell
CD8 Memory_1	T cell
CD8 Memory_2	T cell
CD8 Naive	T cell
cDC2	Mono/DC
gdT	T cell
GMP	Progenitor cells
HSC	Progenitor cells
LMPP	Progenitor cells
MAIT	T cell
Memory B	B cell
Naive B	B cell
NK	NK
pDC	Mono/DC
Plasmablast	B cell
Prog_B 1	Progenitor cells
Prog_B 2	Progenitor cells
Prog_DC	Progenitor cells
Prog_Mk	Progenitor cells
Prog_RBC	Progenitor cells
Treg	T cell

References

[1] Elahi, Z. *et al.* The human dendritic cell atlas: An integrated transcriptional tool to study human dendritic cell biology. *J. Immunol.* **209**, 2352–2361 (2022).

[2] Schmidt, R. *et al.* CRISPR activation and interference screens decode stimulation responses in primary human T cells. *Science* **375**, eabj4008 (2022).

[3] Schmiedel, B. J. *et al.* Impact of genetic polymorphisms on human immune cell gene expression. *Cell* **175**, 1701–1715.e16 (2018).

Table S2 Merging fine cell types in the 10x Multiome dataset to broad ones. We manually merged the fine cell types defined in the 10x Multiome dataset [5] to broad cell types, for benchmark studies on cross-omics annotation (see Section 2.1).

Fine cell types	Broad cell types
B1 B	B cell
CD14+ Mono	Mono/DC
CD16+ Mono	Mono/DC
CD4+ T activated	T cell
CD4+ T naive	T cell
CD8+ T	T cell
CD8+ T naive	T cell
cDC2	Mono/DC
Erythroblast	Progenitor cells
G/M prog	Progenitor cells
HSC	Progenitor cells
ID2-hi myeloid prog	Progenitor cells
ILC	ILC
Lymph prog	Progenitor cells
MK/E prog	Progenitor cells
Naive CD20+ B	B cell
NK	NK
Normoblast	Progenitor cells
pDC	Mono/DC
Plasma cell	B cell
Proerythroblast	Progenitor cells
Transitional B	B cell

[4] Stuart, T. *et al.* Comprehensive integration of Single-Cell data. *Cell* **177**, 1888–1902 (2019).

[5] Luecken, M. *et al.* Vanschoren, J. & Yeung, S. (eds) *A sandbox for prediction and integration of dna, rna, and proteins in single cells.* (eds Vanschoren, J. & Yeung, S.) *Proceedings of the Neural Information Processing Systems Track on Datasets and Benchmarks*, Vol. 1 (2021).
