# Supplementary material for: Φ-Space: continuous phenotyping of single-cell multi-omics data
Source: Genome Biol. 2025 Sep 30;26:323. doi: 10.1186/s13059-025-03755-8 (PMC12482742; doi:10.1186/s13059-025-03755-8)
Supplement: Supplementary file 2 — Additional file 2: Supplementary Methods. Additional details of the $\Phi$Φ-Space method; additional details of the analyses in case studies. [file 13059_2025_3755_MOESM2_ESM.pdf]

Supplementary Methods for "Φ-Space: Continuous phenotyping of single-cell multi-omics data"

Jiadong Mao[1], Yidi Deng[1], Kim-Anh Lê Cao[1,*]

[1]Melbourne Integrative Genomics, School of Mathematics and Statistics, The University of Melbourne, Australia

∗ corresponding author: kimanh.lecao@unimelb.edu.au

S1 Details of Φ-Space

S1 Loss function for parameter tuning

In the Φ-Space framework, each experimental unit (bulk sample or single cell) in the reference is allowed to have multiple class labels (e.g. cell type and sample source). To achieve robust and interpretable continuous phenotyping based on soft classification, we use the residual sum of squares (RSS) to tune the parameters in our PLS model. Given the true $\{-1, 1\}$-valued response matrix Y and a continuous predicted version $\hat{Y}$, the RSS criterion is defined as RSS $= \|Y - \hat{Y}\|_F$, where $\|\cdot\|_F$ denotes the Frobenius norm of matrices (sum of squares of all matrix entries). An increasing number of recent theoretical and empirical machine learning works have suggested that training classification models using RSS tend to yield robust and generalisable results for complex data [1–3].

S2 PLS component and feature selection

For selecting the number of PLS components `ncomp`, we compute a 5-fold cross-validation (CV) version of the RSS criterion, where we split the reference data into training and validation folds. For atlas-scale single-cell references, computing CV is time consuming. Thus, we first use the rule-of-thumb `ncomp` $= K$ for choosing the number of PLS components without any feature selection, as described in Section

5.1. For smaller-scale references such as bulk references, we plot the CV RSS values computed for an equispaced grid of candidate `ncomp` values on the logarithmic scale. Additional file 1: Fig S6A shows such a plot for the bulk reference data [4] used in the DC case study in Section 2.2. We chose `ncomp` = 30 since further increase of `ncomp` does not significantly decrease the RSS.

After selecting `ncomp`, and if feature selection is desired (e.g. for removing platform effects confounding biology as in our DC case study), we perform variable selection to characterise each of the K phenotypes in the reference as follows. We select the top `nfeat` features that are most useful for predicting that phenotype and then choose the union of these K sets of features as the selected features. We then use only the selected features to compute a PLS model. Here the goal is to improve computational efficiency and model interpretability rather than prediction accuracy. To evaluate the contribution of each feature towards predicting a given phenotype, we extract the regression coefficient matrix B of size $(P \times K)$ from the PLS model computed using all P features and a selected `ncomp` value. Let $\tilde{B}$ denote the version of B where each entry is replaced by its absolute value. Now the k-th column of $\tilde{B}$ can be viewed as importance scores for all P features in predicting the k-th phenotype. We then choose the top `nfeat` features with the highest importance scores. Hence we have reduced the problem of feature selection to the selection of `nfeat`, which is again done via examining the RSS plot. Additional file 1: Fig S6B shows such a plot for the bulk reference in the DC case study. We chose `nfeat` = 265 which minimises the RSS value. This gives us a total of 1822 features; see Section 2.2 for more details on an evaluation of these features.

For all the case studies, except for the DC case, we used single-cell reference datasets, which contained large number of cells. For these case studies, it was more time consuming to run the CV algorithm for selecting PLS components and number of features. Instead we used the default setting – a number of PLS components equal to K the total number of phenotypes and used all features. We observed that in practice changing the number of features did not seem to effect the model performance,

unless very strong batch effects are present in the reference data, such as in the DC case. The default choice of PLS components was robust across all case studies.

S3 Normalisation of phenotype space embeddings

Recall that the Φ-Space scores $\hat{Y}_{\text{query}}$ is defined as a normalised version of the raw PLS scores $X_{\text{query}}B$ of size $(M \times K)$ (or $Z_{\text{query}}B_{\text{bridge}}$ for cross-reference transfer). Given a generic matrix A of size $(M \times K)$, we obtain its normalised version $B = \sigma(A)$ by centering each column of A by its median and scaling it by its largest absolute value. This normalisation step effectively improves the prediction for rarer cell types, thus alleviating the so-called 'masking' problem of linear classifiers for data with imbalanced class proportions [5].

S2 Details of case studies

S1 Preprocessing of data

Here we describe how data were processed before applying Φ-Space. See Section 2.1 for a description of data.

Case study 1: DC. Since the bulk reference data [4] have been rank normalised, we also applied rank normalisation to the query scRNA-seq data. Rank normalisation was defined in [6] and we implemented it using the `RankTransf` function in our PhiSpace R package.

Case study 2: Perturb-seq. We downloded the DICE bulk atlas using `celldex::DatabaseImmuneCellExpressionData()`. The downloaded data were TPM normalised and we convert it back to counts using $2^{\text{TPM}} - 1$, and then normalised the counts using scran [7]. For query Perturb-seq data [8], we downloaded Seurat object `HuTcellsCRISPRaPerturbSeq_Re-stimulated.h5Seurat` from Zenodo (https://zenodo.org/records/5784651) and normalised the raw counts using scran [7].

Case study 3: CITE-seq. The RNA modality in both reference and query were normalised using the R package scran [7]. The ADT modality were normalised

using central log ratio (CLR) transform, implemented by the `CLRnorm` function in our PhiSpace R package.

Case study 4: scATAC-seq. The RNA modality of all 13 batches were normalised, batch by batch, using scran [7]. For the two representation of ATAC modality: peaks were normalised, batch by bach, using term-frequency inverse-document-frequency as implemented by the `RunTFIDF` function in the R package Signac; gene activity scores were normalised, batch by batch, using scran.

S1.1 Alignment score

We computed the alignment score defined in [9], using the R implementation `alignment_score` in the `PLSDAbatch` package [10]. The `alignment_score` function returns a score between 0 and 1 measuring how well cells across different groups (e.g. cell types, batches) are mixed: the score equals to 0 if the nearest neighbours of each cell are all from the same condition as that cell, i.e. perfect separation of conditions.

S1.2 PCA-based reference mapping

To obtain Fig 3A, we first computed the leading PCs of the bulk reference gene expression matrix, using only the genes selected by Φ-Space . We then computed the embedding of the query single cells by projecting the scRNA-seq data to the reference PC1 and PC2, using the loadings computed using the bulk reference.

S2 Definition of Φ-Space-derived CD4 and T cell activation scores.

Φ-Space-derived CD4 and T cell activation scores featured in the Perturb-seq case study were defined based on the phenotype space embedding (Additional file 1: Fig S4). To compute the CD4 score of a cell, we first compute AveCD4, the average score of all CD4 subtypes (e.g. 'T cells, CD4+, memory TREG'), and AveCD8 the average score of all CD8 subtypes (e.g. 'T cells, CD8+, naive'). Then the CD4 score of a cell is defined as $(\text{AveCD4} - \text{AveCD8})^{1/3}$, where the cube root transformation is to make

the distribution of (AveCD4 − AveCD8) less skewed. The activation score of a cell is simply defined as the cell's Φ-Space scores of 'T cells, CD4+, naive, stimulated' and 'T cells, CD8+, naive, stimulated'.

S2.1 Implementation of alternative classification methods

In both the CITE-seq and the scATAC-seq case studies (Sections 2.4 and 2.5), we used the R package Seurat [11]. In addition, in the scATAC-seq case study we also implemented SingleR [12] and scANVI [13, 14] using the default tuning parameters used in their vignettes. To make the results more comparable, we used scran [7] as the normalisation method for all three methods.

- Seurat V3. We followed the Seurat vignette [15]. We skipped the normalisation step using `NormalizeData` and instead included scran normalised data as the `data` slot in the Seurat object. We used the predicted cell type scores as the Seurat V3 phenotype space embedding in the CITE-seq case study, and the predicted cell types in the scATAC-seq case study.
- Seurat V4. We used Seurat V4 only for the CITE-seq case study. We followed a similar workflow as Seurat V3, with additional steps for weighted nearest neighbour integration of the RNA and ADT modalities in the reference data. Then the RNA and ADT modalities in the query were mapped to the integrated reference to derive the Seurat V4 phenotype space embedding.
- SingleR. We used `trainSingleR` and `classifySingleR` in the R package SingleR with default parameters. Since SingleR does feature selection implicitly, we did not select highly variable genes (HVGs) before training the model.
- scANVI. We used scANVI as implemented in python library scArches [14]. R package reticulate was used to call python functions from R. We followed the scANVI vignette [16]. We used Seurat to select HVGs before training scANVI, so that scANVI used the same HVGs as our implementation of Seurat cell typing above.

In both the scATAC-seq case studies (Section 2.5), for transferring cell types from scRNA-seq reference to scATAC-seq query, we implemented Seurat bridge integration

(SeuBI; [17]) by following the SeuBI vignette [18]. We used SCTransform as suggested by that vignette.

S2.2 Classification errors

We calculated both overall and balanced classification errors to evaluate the label transfer results. Only balanced error were shown in the main manuscript since the overall error led to the same conclusion as in the scATAC-seq case study (Section 2.5). In particular, given predicted cell type labels $\{\hat{l}_1, \dots, \hat{l}_n\}$ and ground truth cell type labels $\{l_1, \dots, l_n\}$, where each $l_i \in \{1, \dots, K\}$ for $i = 1, \dots, n$. Then the overall classification error is defined by $(1/n) \sum_{i=1}^{n} \mathbf{1}(\hat{l}_i \neq l_i)$, where $\mathbf{1}(\cdot)$ denote the indicator function. The balanced classification error is defined by $(1/K) \sum_{k=1}^{K} \big\{(1/n_k) \sum_{i=1}^{n} \mathbf{1}(l_i = k) \mathbf{1}(\hat{l}_i \neq l_i)\big\}$, where $n_k = \sum_{i=1}^{n} \mathbf{1}(l_1 = k)$ denotes the number of observations from the k-th cell type.

S2.3 Details of phenotype and omics space clustering

For phenotype space clustering, we concatenated the 22-dimensional phenotype space embeddings of cells from different query batches and applied k-means clustering by using the R function `kmeans` with the `algorithm="Lloyd"`, `iter.max=500` and `nstart=50`. The same k-means implementation was used for omics space clustering below.

For omics space clustering, we concatenate the normalised peaks or gene expressions from query batches and then computed dimension reduction. For peaks, we computed singular value decomposition (SVD) and used the 2nd to the 23rd SVD components as the input to k-means, to match the dimensionality of phenotype space embeddings. Discarding the first SVD component is recommended by the Seurat workflow since this component is usually heavily contaminated by technical noise [18]. For concatenated GA scores, we computed the top 22 PCs, which were then used as input to k-means.

S2.4 Metrics for clustering

We computed adjusted Rand index, normalised mutual information using the `clustComp` function in R package aricode [19]. We used our own R implementation of Van Rijsbergen's F measure [20], which is robust version of the commonly used purity metric; see [21] for the definition of the F measure and its superiority compared to purity.

References

[1] Beleites, Claudia and Salzer, Reiner and Sergo, Valter. Validation of soft classification models using partial class memberships: An extended concept of sensitivity & co. applied to grading of astrocytoma tissues. *Chemometrics and Intelligent Laboratory Systems* **122**, 12–22 (2013).

[2] Hui, L. & Belkin, M. Evaluation of neural architectures trained with square loss vs Cross-Entropy in classification tasks. *arXiv* (2020). URL https://arxiv.org/abs/2006.07322.

[3] Hu, T., Wang, J., Wang, W. & Li, Z. Understanding square loss in training overparametrized neural network classifiers. *NeurIPS* **35**, 16495–16508 (2022).

[4] Elahi, Z. *et al.* The human dendritic cell atlas: An integrated transcriptional tool to study human dendritic cell biology. *J. Immunol.* **209**, 2352–2361 (2022).

[5] Hastie, T., Tibshirani, R. & Friedman, J. *The Elements of Statistical Learning* (Springer New York, 2009).

[6] Angel, P. W. *et al.* A simple, scalable approach to building a cross-platform transcriptome atlas. *PLoS Comput. Biol.* **16** (2020).

[7] Lun, A. T. L., Bach, K. & Marioni, J. C. Pooling across cells to normalize single-cell RNA sequencing data with many zero counts. *Genome Biol.* **17** (2016).

[8] Schmidt, R. *et al.* CRISPR activation and interference screens decode stimulation responses in primary human T cells. *Science* **375**, eabj4008 (2022).

[9] Butler, A., Hoffman, P., Smibert, P., Papalexi, E. & Satija, R. Integrating single-cell transcriptomic data across different conditions, technologies, and species. *Nat. Biotechnol.* **36**, 411–420 (2018).

[10] Wang, Y. & Lê Cao, K.-A. PLSDA-batch: a multivariate framework to correct for batch effects in microbiome data. *Brief. Bioinform.* **24** (2023).

[11] Stuart, T. *et al.* Comprehensive integration of Single-Cell data. *Cell* **177**, 1888–1902 (2019).

[12] Aran, D. *et al.* Reference-based analysis of lung single-cell sequencing reveals a transitional profibrotic macrophage. *Nat. Immunol.* **20**, 163–172 (2019).

[13] Xu, C. *et al.* Probabilistic harmonization and annotation of single-cell transcriptomics data with deep generative models. *Mol. Syst. Biol.* **17** (2021).

[14] Lotfollahi, M. *et al.* Mapping single-cell data to reference atlases by transfer learning. *Nat. Biotechnol.* **40**, 121–130 (2022).

[15] Satija Lab. Mapping and annotating query datasets (2024). URL https://satijalab.org/seurat/articles/integration_mapping.

[16] Yosef Lab. Semi-supervised surgery pipeline with scanvi. *scANVI Package Vignette* (2024). URL https://docs.scarches.org/en/latest/scanvi_surgery_pipeline.html.

[17] Hao, Y. *et al.* Dictionary learning for integrative, multimodal and scalable single-cell analysis. *Nat. Biotechnol.* **42**, 293–304 (2024).

[18] Satija Lab. Dictionary learning for cross-modality integration. *Seurat Package Vignette* (2024). URL https://satijalab.org/seurat/articles/seurat5_integration_bridge.

[19] Chiquet, J., Rigaill, G. & Sundqvist, M. aricode: Efficient computations of standard clustering comparison measures. *GitHub* (2024). URL https://github.com/jchiquet/aricode. R package version 1.0.3.

[20] Van Rijsbergen, C. J. Foundation of evaluation. *Journal of Documentation* **30**, 365–373 (1974).

[21] Amigó, E., Gonzalo, J., Artiles, J. & Verdejo, F. A comparison of extrinsic clustering evaluation metrics based on formal constraints. *Inf. Retrieval* **12**,

461–486 (2009).
